# Genetic drift and bottleneck do not influence diversity in Toll‐like receptor genes at a small spatial scale in a Himalayan passerine

**DOI:** 10.1002/ece3.6855

**Published:** 2020-10-15

**Authors:** Mridula Nandakumar, Farah Ishtiaq

**Affiliations:** ^1^ Centre for Ecological Sciences Indian Institute of Science Bangalore India; ^2^ Present address: Department of Biology Lund University Lund Sweden

**Keywords:** *Aegithalos concinnus*, *Aegithalos niveogularis*, Bottleneck, genetic variation, microsatellites, pathogen‐mediated selection, toll‐like receptors, western Himalaya

## Abstract

Genetic diversity is important for long‐term viability of a population. Low genetic diversity reduces persistence and survival of populations and increases susceptibility to diseases. Comparisons of the neutral markers with functional loci such as immune genes [Toll‐like receptors; TLR] can provide useful insights into evolutionary potential of a species and how the diversity of pathogens and selection pressures on their hosts are directly linked to their environment. In this study, we compare genetic diversity in neutral (eleven microsatellite loci) and adaptive (seven TLR loci) loci to determine genetic variation in a nonmigratory western Himalayan passerine, the black‐throated tit (*Aegithalos concinnus*), distributed across an elevation gradient with varying degree of pathogen‐mediated selection pressure. We further compare the diversity in TLR loci with a high‐elevation sister species, the white‐throated tit (*Aegithalos niveogularis*). Our results indicate a lack of population genetic structure in the black‐throated tit and signatures of a past bottleneck. In contrast, we found high diversity in TLR loci and locus‐specific (TLR7) signatures of pathogen‐mediated selection, which was comparable to diversity in the white‐throated tit. Levels of diversity at TLR5 locus corresponded very closely with neutral microsatellite variation. We found evidence of positive selection in TLR1LA, TLR5, and TLR7 loci highlighting the importance in pathogen recognition. Our finding demonstrates that reduction in neutral variation does not necessarily lead to reduction in functional genetic diversity and probably helps in revival of population in a widespread species.

## Graphical abstract

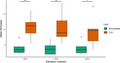

## 1 INTRODUCTION

Genetic diversity is important for survival and persistence of wild populations—high level of genetic variation may allow for flexibility to adapt through changing environments (Booy et al., 2000; Lacy, 1997; Markert et al., 2010; Yildrim et al., 2018). Comparison of neutral loci with adaptive loci provides critical information on adaptive evolutionary potential of a species to environmental changes and extinction risk (Agudo et al., 2012; Dionne et al., 2007; Frankham et al., 1999; Hartmann et al., 2014; Meyer‐Lucht et al., 2016). Mismatch in genetic diversity patterns between neutral and adaptive loci can be influenced by selective forces. For example, the selection pressure driven by pathogens is strong on functional loci and significantly influences genetic diversity at specific loci compared to effects on the neutral genetic diversity. Furthermore, both neutral and functional genetic diversity are affected by random fluctuations in the number of alleles due to genetic drift, population bottleneck, demographic history, and gene flow in a population (Takahata, 1990; Takahata & Nei, 1990; Frankham, 1996; Weber et al., 2004; Grueber, Wallis, & Jamieson, 2013; Gonzalez‐Quevedo et al., 2015; Cortázar‐Chinarro et al., 2017). Therefore, quantification of genetic diversity provides valuable insights on how ecological and evolutionary forces shape the genetic diversity in widespread species as well as species of conservation concern.

In vertebrates, maintenance of immunity is a complex process driven by innate and adaptive immune responses. The toll‐like receptors (TLRs) are an important component of the innate immune system that plays an important role in recognizing pathogens and lead the initial fight against infections (Akira et al., 2001). The TLRs are a family of evolutionarily conserved genes, belonging to the pattern‐recognition receptor (PRRs) family, that encode immune cell receptors known to recognize specific molecular ligands present in pathogens, termed pathogen‐associated molecular patterns (PAMPS) (Akira et al., 2001; Keestra et al., 2013). By means of cellular cascades upon TLR activation, the adaptive immune system is subsequently triggered through antigen presentation to T cells by the Major Histocompatibilty Complex (MHC) genes (Iwasaki & Medzhitov, 2015). Species that experience a change in pathogen pressure evolve a high MHC diversity that helps to identify and eliminate a broad spectrum of pathogens (Alcaide et al., 2014; Westerdahl et al., 2004). Nonetheless, the studies involving the MHC are limited by technical challenges, especially in the case of passerines, where a high rate of gene duplication has been recorded (Minias et al., 2019). More importantly, the variations in the MHC genes alone are incapable of explaining differences in immunity, due to the collective nature of immune response, whereby different molecular players interact with the pathogen over the course of an infection (Acevedo‐Whitehouse & Cunningham, 2006).

The relatively simple genetic structure, few pseudogenized and duplicated genes, and importance in being first responders to infections have led to increased emphasis on TLR genes in a wide range of taxa, including ungulates, birds, cetaceans, and wild rodents (Grueber et al. 2013; Ishengoma & Agaba, 2017; Tschirren et al., 2013). In birds, the TLR repertoire is made up of ten genes, with the gene family observed to have evolved by ancient duplication events (e.g., TLR1LA and TLR1LB, TLR2A and TLR2B; Temperley et al., 2008; Yilmaz et al., 2005). However, more recent duplication and pseudogenization events in TLR7 and TLR5, respectively, and gene conversion in two paralogues—TLR1LA and TLR1LB, TLR2A and TLR2B—have occurred independently in several passerine species (Velová et al., 2018). Distinct TLR variants have shown to be associated with diseases (Netea et al., 2012), with certain variations serving as good indicators of infection status (e.g., Borrelia infection in bank voles; Tschirren et al., 2013), disease severity (in humans, Leoratti et al., 2008) as well as survival (e.g., New Zealand robin; Grueber et al., 2012).

Parasite‐mediated selection pressure can alter the diversity and allele frequencies of immune genes in many ways. For example, the TLR genes interacting directly with pathogens are often under positive selection (e.g., Alcaide et al., 2014; Darfour‐Oduro et al., 2015; Tschirren et al., 2011). While purifying selection is experienced across all loci, episodic positive selection has played an important role in the evolution of most avian TLRs (Grueber, Wallis, & Jamieson, 2014; Velvová et al., 2018). Therefore, positive selection mediated by pathogens on immune genes plays an important role in shaping co‐evolutionary dynamics between the host and pathogen (Antonides et al., 2019; Babik et al., 2014; Cornetti et al., 2018; Shultz & Sackton, 2019). However, in some human populations, balancing selection is the main force in shaping the evolution of immunity genes (Ferrer‐Admetlla et al., 2008).

The diversity of pathogens and selection pressures on their hosts are directly linked to environment and can vary even at a small spatial scale (e.g., Cornetti et al., 2018; Gonzalez‐Quevedo et al., 2016). In the context of spatial variation, elevational changes in temperature alter parasite transmission dynamics (Baur & Baur, 2005; Ishtiaq & Barve, 2018; Muths et al., 2008; Tarageľová et al., 2016; Zamora‐Vilchis et al., 2012) and thereby pathogen‐mediated selection pressure on host immune genes (Cornetti et al., 2018). Birds are natural reservoirs of a diverse community of pathogens and often harbor hemosporidian parasites (Hernández‐Lara et al., 2017; Ishtiaq et al., 2017a; Krone et al., 2008; Loiseau et al., 2010). While many studies linked TLR variation within bottlenecked populations (Gonzalez‐Quevedo et al., 2015; Grueber et al., 2012; Hartmann et al., 2014), none have evaluated TLR evolution with pathogen risk across an elevational gradient in birds (but see Cornetti et al., 2018). The western Himalayan montane system is a species‐rich highly seasonal ecosystem (Srinivasan et al., 2018). The risk of *Plasmodium* and *Haemoproteus* infections in the western Himalayan birds is highest at the low elevations and decreases with increasing elevation whereas *Leucocytozoon* infection risk increases with elevation (Ishtiaq & Barve, 2018). Therefore, the western Himalayan birds provide a fantastic opportunity to explore the extent of functional gene diversity distributed across an elevational gradient.

In this study, we compare patterns in neutral and functional diversity in a western Himalayan passerine—the black‐throated tit [*Aegithalos concinnus iredalei*, a 5–7 gm passerine, common and widely distributed in Pakistan, Nepal, Bhutan, India, Myanmar, Thailand, Laos, Vietnam, and mainland China, but see recent split as *Aegithalos iredalei*; del Hoyo et al., 2020]. The black‐throated tit is a nonmigratory (sedentary) species distributed in lower to midelevation (1,800–2,600 m a.s.l.) woodland forest. The black‐throated tit is a co‐operative breeder favoring male‐biased dispersal (Dai et al., 2013; Li et al., 2012). However, there are no studies conducted on population dispersal or gene flow in India. Using eleven neutral and seven adaptive loci in the black‐throated tit, we aim to understand evolution of genes at three levels: First, we quantify and compare TLR variation between the black‐throated tit and a close congeneric, ecologically similar species in the western Himalaya, the white‐throated tit [*Aegithalos niveogularis*; a 7–8 gm passerine with restricted range in the western Himalayas‐ mainly Pakistan and India, Harrap, 2020]. The white‐throated tit is a sedentary high‐elevation species (2,800–3,200 m) but not found in sympatry with the black‐throated tit. Owing to spatial variation in pathogen‐mediated selection pressure, we predict that black‐throated tit populations will possess a diverse set of TLR genes compared to their high‐elevation counterpart, the white‐throated tit. We compare signatures of selection at the haplotype and protein levels for each species. Second, we explore elevational variation in TLR haplotypes and malaria infection risk in the black‐throated tit. Third, we quantify neutral variation in the black‐throated tits across an elevational gradient and explore pathogen‐mediated selection by comparing neutral versus TLR variation in the black‐throated tit.

We explore the role of drift and signatures of genetic bottleneck in shaping the neutral genetic diversity. In addition, we compared the patterns in neutral variation in black‐throated tit in India with the genotype data (using similar neutral loci) of a closely related congeneric species, *Aegithalos concinnus talifuensis* that is widespread across mainland China and recently split as separate species *A. concinnus* (Dai et al., 2017). These data allowed us to compare signatures of bottlenecks, effect of geographical distance on genetic differentiation, and population structure in two regional populations on a similar spatial scale.

## 2 MATERIALS AND METHODS

### 2.1 Bird blood sampling

**FIGURE 1:**
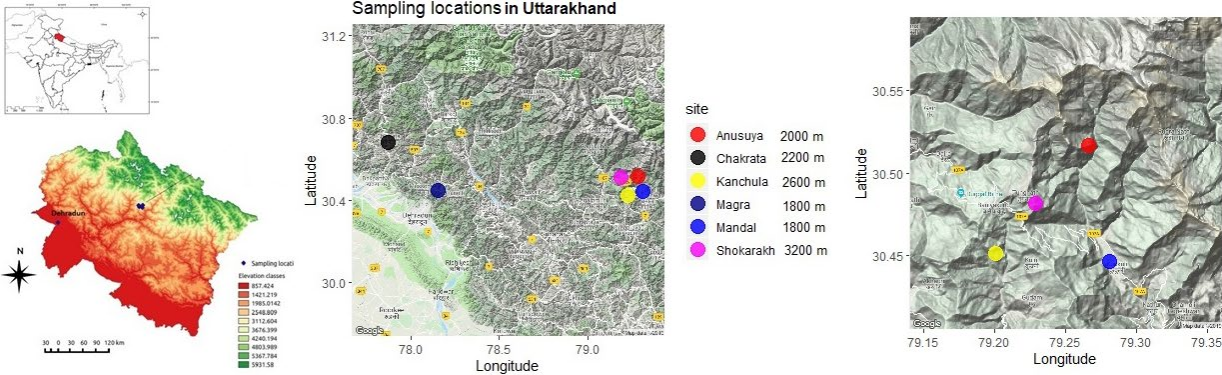
Map showing sampling locations for the black‐throated tit (1,800–2,600 m) and white‐throated tit (3,200 m) in Uttarakhand, India

Blood samples were collected between 2009 and 2016 during the breeding season (April‐May) using mist nets in the Garhwal region, Uttarakhand, India, in the western Himalaya. The black‐throated tits were sampled from five sites spanning an elevational gradient from 1,800–2,600 m a. s. l. The distance between sampled locations ranged from 5 to 150 km. The white‐throated tits were sampled from one location at 3,200 m a.s.l. (see details in Table 1 and Figure 1). At each sampling site, 6–12 mist nets were set up in high bird activity locations. Each bird caught was identified and ringed with a numbered metal ring. Blood was drawn from the sub‐brachial vein (blood collected never exceeded 10% of body weight) and stored in SET buffer (20–40 μL blood in 500 μL buffer 0.15 M NaCl, 0.05 M Tris, 0.001 M EDTA, pH 8.0) for subsequent analysis. Captured individuals were released at the site immediately after processing.

**TABLE 1:** Sample collection details on elevation, species, sample size, and geographic co‐ordinates in the western Himalaya

Sampling site	Elevation (m a.s.l.)	Elevation category	Species	Sample size	Latitude	Longitude
Mandal (MAN)	1,800	Low	BTT	10	30.44685	79.27328
Magra (MAG)	1,800	Low	BTT	4	30.45587	78.16158
Anusuya (ANU)	2,000	Medium	BTT	41	30.47888	79.28503
Chakrata (CHA)	2,200	Medium	BTT	4	30.702	77.869
Kanchula (KAN)	2,600	High	BTT	19	30.45913	79.22744
Shokarakh (SHO)	3,200	High	WTT	8	30.4786	79.21798

Abbreviations: BTT, black‐throated tit; WTT, white‐throated tit.

### 2.2 Molecular methods

#### 2.2.1 Quantification of TLR variation in the black‐throated tit and white‐throated tit

Genomic DNA was extracted by phenol–chloroform method (Sambrook & Russell, 1983). We amplified seven avian‐specific TLR loci—TLR1LA, TLR1LB, TLR2, TLR3, TLR4, TLR5, TLR7—an average of 850 bp targeting the extracellular leucine‐rich repeat region of each gene as described in Alcaide and Edwards (2011). The TLR2 primer amplified only TLR2A inconsistently, but not TLR2B. Therefore, we designed species‐specific primers for TLR2A (aegiTLR2; Table S1) using Primer3web v 4.1.0 (Untergasser et al., 2012) and checked for specificity using NCBI’s Primer‐BLAST. These primers amplified TLR2A in the white‐throated tit as well. We could not amplify TLR15 and TLR21 using alternate primers as well (Grueber & Jamieson, 2013). PCRs were set up in 25 μL reaction volumes containing a final concentration of 1× Taq Buffer, premixed with 1.5 mM MgCl2 (New England Biolabs, Ipswich, MA, USA), 0.025 μM of each dNTP, 0.6 mM of forward and reverse primer each, 2.5 units of Taq polymerase and *c*. 10 ng/μL of template genomic DNA. The PCR conditions were as follows: initial denaturation for 3 min, followed by 35 cycles of denaturation at 94°C for 40 s, locus‐specific annealing for 40 s (supplementary Table S1 for details), extension at 72°C for 80 s and completed with a final extension step of 72°C for 10 min. PCR amplifications were checked on a 1.5% agarose gel and visualized with SYBR Safe DNA Gel stain (Invitrogen, Waltham, MA, USA). PCR products were cleaned by using Qiagen's PCR Purification kit (Qiagen, Hilden, Germany) and were sequenced in both directions. Sequences were analyzed using Geneious R9.1.5 and standard International Union of Pure and Applied Chemistry (IUPAC) recommended codes for degenerate nucleotides were introduced for heterozygous sites detected in chromatograms. We repeated PCRs and sequenced samples with ambiguous chromatogram peaks. Diploid genotypes were resolved into individual haplotypes using the Bayesian PHASE algorithm (Stephens & Donnelly, 2003) implemented in DnaSP v6 (Rozas et al., 2017) with default settings of 100 iterations, a thinning interval of 1, and 100 burn‐in iterations. We calculated basic polymorphism statistics such as the number of polymorphic sites (number of segregating sites), number of haplotypes, haplotype and nucleotide diversity, and average number of nucleotide changes using DnaSP. Deviations from neutrality at the nucleotide level were tested with Tajima's D, Fu and Li's D, and Fu and Li's F. Deviations from HWE were tested using Genepop v4.2 online. TLR sequences were submitted with accession number MN629487‐MN646077 in GenBank database (Table S2).

### 2.3 Comparison of TLR allelic richness at nucleotide and protein levels

We compared allelic richness at both nucleotide and protein levels. To understand selection pressure at the protein level we translated the nucleotide sequences for each TLR locus into protein sequences using the “*Biostrings”* package in R (Pagès et al., 2019).

Allelic diversity metrices are sensitive to sample size, so we used the Chao richness index and rarefaction curves to estimate allelic richness at nucleotide level by each locus. We calculated allelic richness after correcting for differences in sample sizes using the lowest sample size for any loci (from rarefaction curves) by species and elevation, thereby avoiding data extrapolation using the software HP‐RARE v1.0 (Kalinowski, 2005). We compared estimated allelic richness in TLRs in the black‐throated tit at three levels: (a) neutral loci versus TLR loci (adjusted for sample size) across elevation (low, medium or high); (b) pairwise comparisons in TLR allelic richness for each elevation category; (c) pairwise comparisons of allelic richness in the black‐throated tit by elevation with sister species—the white‐throated tit. We used Welch *t* test to test significance of unequal variances between two means (Welch, 1951).

### 2.4 Haplotype network construction

We constructed haplotype networks of protein sequences for each locus to define genetic clusters using minimum spanning trees built in Arlequin v3.5 (Excoffier & Lischer, 2010). The networks were visualized and their modularity function in Gephi (Bastian et al., 2009) was used to identify the clusters.

### 2.5 Gene conversion in paralogues

Among avian TLRs, gene conversion has been observed between paralogues TLR1LA–TLR1LB and TLR2A–TLR2B (Velvová et al., 2018). We statistically tested for gene conversion events between TLR1LA and TLR1LB in both tit species using the GENECONV v1.81 (Sawyer, 1989). The program was run using the default settings of 10,000 permutations and mismatch set to zero.

### 2.6 Tests for positive selection

Positive selection acting on coding sequences of TLRs was examined by estimating ω (*dN/dS*, the ratio of the rate of nonsynonymous (dN) to the synonymous (dS) substitution per site) using the maximum likelihood‐based method in CODEML program implemented in the Phylogenetic Analysis by Maximum Likelihood (PAML v4.9; Yang, 2007). We built phylogenetic trees for each TLR loci sequenced in both *Aegithalos* species as input tree for using the Jukes‐Cantor substitution model in MEGA X (Kumar et al., 2018). The site models allow the ω ratio to vary among sites (Nielsen & Yang, 1998; Yang et al., 2000). CODEML implements various evolutionary models to determine selection. We used Model M0, the null model which assumes a constant ω rate along all sites. Model M1a assumes neutral evolution, with two possible ω values (ω < 1 and ω = 0 indicating purifying selection and neutral selection, respectively). The model M2a is a positive selection model with an additional value of ω > 1 indicating positive selection. The M7 and M8 models are similar to the M1a and M2 models, respectively, but additionally assume beta distribution of ω. This limits ω values between 0 and 1 for the neutral model, with an additional parameter of ω > 1 for the selection model. The two nested models, M1a/M2a and M7/M8 determine the presence of positive selection. The initial ω value was fixed to 1 for all the models, with an F3x4 codon frequency model, assuming an individual frequency for each nucleotide position in the codon. Likelihood ratio tests (LRT) were then used to conclude any significant differences between the neutral and selection models, based on a chi‐square distribution of data. We used the Bayes Empirical Bayes (BEB) (Yang et al., 2005) analysis to detect the positively selected codons as it accounts for sampling errors. An individual locus was considered to have undergone positive selection (ω > 1) if the posterior probability was ≥0.95. CODEML results could only be predicted for TLR1LA, TLR5, and TLR7 in the white‐throated tit since the other loci had no nonsynonymous changes to predict ω.

To further validate results from CODEML and to account for model‐specific biases, we employed an additional method for predicting episodic and pervasive selection using the Mixed Effects Model of Evolution (MEME) (Murrell et al., 2012) built into Datamonkey 2.0 (Weaver et al., 2018) for the HyPhy package (Pond et al., 2005). Since this model takes into account recombination effects for predicting positively selected sites, we detected the presence of recombination sites in TLR loci by using the genetic algorithm for recombination detection (GARD) (Kosakovsky Pond et al., 2006) built into Datamonkey 2.0. Following the detection of recombinant sites, we used the partitioned data as input for MEME. This is a branch‐site model for selection and predicts two values of dN (β^−^ common to both the neutral and selection model and β^+^ is for the selection model alone) for every value of dS (α). Positively selected sites are determined when β^+^ values exceed α values significantly, as determined by LRT. Loci (TLR1LB, TLR2, TLR3) with less than 3 unique sequences in the white‐throated tit were not used in this analysis.

### 2.7 Molecular sexing and parasite screening in the black‐throated tit

*Aegithalos* species do not exhibit sexual dimorphism. Birds were sexed by amplifying the intronic region of the CHD1Z/W gene using the primers 0057F/002R following PCR conditions as described in Round et al. (2007). We screened samples for infection with three blood parasite genera (*Plasmodium*, *Haemoproteus*, and *Leucocytozoon*) by amplifying a partial fragment 476 bp of the cytochrome *b* gene as described in Hellgren et al. (2004). The infection status was confirmed by sequencing the PCR product bidirectionally.

### 2.8 Spatial variation in TLR haplotypes and infection risk in the black‐throated tit

We used generalized linear model (GLM) to understand the association between TLR haplotypes, TLR haplotype clusters, and TLR cluster zygosity with infection status, sex, and elevation. First, we tested if the presence of TLR haplotypes by loci varied across elevation and sex which were modeled as a fixed effect. Second, we tested if the distribution of the two codon forms in positively selected sites (as identified by CODEML and MEME selection methods) were influenced by elevation and sex, modeled as fixed effects. Finally, we tested if infection status, as a binary response variable, was influenced by TLR haplotypes, elevation, and sex modeled as fixed effects. We assigned TLR haplotypes into clusters using haplotype network analysis (Figures S1–S7). Thus, for each TLR locus, all haplotypes were grouped in 2–5 clusters and association of infection status was then carried out in two ways: i) with specific TLR haplotype clusters or ii) with TLR cluster zygosity, encoded as 0 (homozygous) or 1 (heterozygous) if the same or different haplotype cluster was present, respectively. We fitted ten GLMs based on the number of clusters present for the TLR locus and six models with cluster heterozygosity. We used the MuMIN package (Bartoń, 2013) to identify the best model based on quasi small‐sample corrected Akaike information criterion (qAIC_c_). Models with a difference (ΔAICc) of ≤2 are as parsimonious as the best‐fit model (lowest AICc) and are considered strong evidence that the quality of the candidate models differs from one another (Burnham & Anderson, 2002) and the confidence interval function was used to determine the 95% confidence intervals of estimated parameters. Models were considered significant if the upper or lower confidence interval did not span zero. All GLM analyses were carried in the lme4 package (Bates et al., 2015) using a binomial error distribution and logit link function. In models that showed overdispersion of data, quasibinomial distribution was used to correct this effect. The significance of the models was tested using Wald's chi‐square test (Bolker et al., 2009).

### 2.9 Neutral variation in the black‐throated tit

We used eleven putatively neutral microsatellites—TG01040, TG03031, TG04004, TG04041, TG01147 (Dawson et al., 2010), and Ase18, Ase37, Escµ6, Man13, Pca3, and PmaD22 (Simeoni et al., 2007), to characterize the population structure in black‐throated tit populations. These markers have been used in determining neutral variation in the black‐throated tit populations in China (Dai et al., 2017). Furthermore, using similar markers allowed us to compare the genetic structure in two populations (see below in Population genetic structure). We multiplexed loci in four replicates (to account for allele dropout) with each locus tagged with fluorescently labeled dye (6‐Fam, VIC or NED) at the 5’ end of the forward primer (Table S3 for details). The total volume of each multiplex reaction was 10 μL containing 4 μL Multiplex PCR Master Mix (Qiagen, Hilden, Germany), 1 μL Q‐solution, final primer concentration for each loci (Table S3), and *c*. 10 ng/μl of DNA template, with the remaining volume made up with water. The PCR conditions used were: initial denaturation at 94°C for 15 min, followed by 30 cycles of denaturation at 94°C for 30 s, annealing at 58°C for 90 s, extension at 72°C for 45 s, and completed with a final extension step at 60°C for 45 min. PCR amplifications were visualized on a 1.5% gel stained with SYBR Safe DNA gel stain (Invitrogen, Waltham, MA, USA) and bright bands were considered for genotyping (Eurofins Genomics, Bengaluru, India). Alleles were scored manually using Geneious v 9.1.5 (https://www.geneious.com).

### 2.10 Quantification of neutral variation in the black‐throated tit

All analyses were conducted in R v3.5.3 (R Core Team 2017) unless stated otherwise. The genotyping errors as null alleles and allele drop out were detected using MICRO‐CHECKER v2.2.3 (Oosterhout et al., 2004). Estimates of the number of alleles, observed, expected heterozygosity, and polymorphism information content (PIC) were calculated with CERVUS v3.0.7 (Kalinowski et al., 2007). All populations were tested for Hardy–Weinberg equilibrium (HWE) and linkage disequilibrium using Genepop v4.2 online (http://genepop.curtin.edu.au/genepop_op1.html) with default values and the p‐values corrected using Benjamini–Hochberg false discovery rate (FDR) which is a powerful tool that decreases the false discovery rate (Benjamini & Hochberg, 1995). Pairwise *F_ST_* values between sampling sites were determined in Arlequin v3.5. Inbreeding coefficient *F_IS_* and 95% confidence interval were calculated for each population using the package diveRsity (Keenan, McGinnity, Cross, Crozier, & Prodöhl, 2017).

### 2.11 Population genetic structure in the black‐throated tit

We used five methods to assess population structure in the black‐throated tit. The first two clustering methods used a Markov chain Monte Carlo (MCMC) algorithm, built‐in STRUCTURE v2.3.4 (Pritchard et al., 2000), and the discriminant analysis of principal components (DAPC) (Jombart et al., 2010). In addition, three distance‐based methods were used: *F_ST_* estimations, the analysis of molecular variance (AMOVA), and isolation by distance (IBD).

Using the Bayesian clustering method STRUCTURE, we explored the possibility of each individual sample belonging to one of several clusters (K) based on allele frequencies. We ran *K* = 1 to *K* = 10, for twenty independent iterations with 500,000 MCMC steps, burn‐in of 50,000, using an admixture model having correlated allele frequencies. We used Structure Harvester (Earl & vonHoldt, 2012) to determine best K following Evanno et al. (2005). CLUMPAK (Kopelman et al., 2015) was used to visualize STRUCTURE results. For populations with high levels of gene flow or low population differentiation (*F*_ST_ < 0.03), STRUCTURE often fails to accurately assign individuals to discrete populations (Latch et al., 2006). Therefore, DAPC was performed using “*adgenet”* package in R (Jombart, 2008). We used PCA value of five, which successfully categorized the majority of the data with a confident classification of individuals into separate clusters by imposing a filter of membership probability *q* > 0.7.

We used AMOVA to check for genetic structuring within the population, by specifying individuals from genetic clusters as predicted by DAPC in Arlequin.

To understand the relationship between spatial patterns with genetic differentiation, Slatkin's linearized *F*_ST_ (*F*_ST_/1−*F*_ST_) obtained for neutral loci were correlated against log‐transformed geographical distances between sampling locations using a Mantel test with 16,000 permutations in Arlequin. Geographic distances were calculated using the R package “*geosphere”* (Hijmans, Williams & Vennes, 2017).

### 2.12 Comparison of genetic variation in the black‐throated tit across two regions: India and China

To accurately compare the spatial structure and population genetic variation in the present distributional range of the species, 78 samples of *Aegithalos concinnus talifuensis* populations in mainland China (Dai et al., 2017) were randomly sampled from the genotype data. We retained monomorphic loci (TG01040, TG04041, Man13) in our dataset to account for heterozygosity loss in our data. We compared these data at two levels:

#### 2.12.1 Spatial autocorrelation

We examined the effect of geographical distance on the genetic differentiation between individuals in each population by region. Regression was performed for developing an average kinship coefficient (F*ij;* Loiselle et al., 1995) between pairs of individuals that were closer together (the first distance interval, ~5 km), and the logarithm of geographic distance was conducted in SPAGeDi v 1.2 (Hardy & Vekemans, 2002). Estimates close to 1 and −1 indicate high and low relatedness, respectively, and values close to zero indicate no relationship between relatedness and distance class.

#### 2.12.2 Population bottleneck

There was no evidence of population structure, so we combined all populations by regions to test for genetic bottlenecks. We used two methods to test for signatures of population bottleneck at neutral loci. First, we calculated *M*, a ratio based on the number of alleles to the allele size range (Garza & Williamson, 2001) in Arlequin to detect signatures of any past genetic bottleneck. The second method was used to detect recent bottlenecks using stepwise mutation model (SMM) and two‐phase model (TPM), both run at variance = 12, probability = 90% and for 10,000 replicates in BOTTLENECK v1.2 (Piry et al., 1999). Significance of the models was assessed using the sign test and Wilcoxon signed‐rank test for heterozygosity excess and the shape of the distribution curve indicating bottleneck was noted.

## 3 RESULTS

### 3.1 TLR variation in the black‐throated and white‐throated tits

For functional genetic diversity, we amplified 70–78 samples of the black‐throated tit for TLR loci and 6–8 samples of the white‐throated tit (Table 2; Tables S4 and S5). For the black‐throated tit populations, an average of 13 polymorphisms resulted in a mean of 38 haplotypes across seven loci (Table S6). Haplotype and nucleotide diversity varied between 0.56–0.99 and 0.0007–0.004, respectively. However, in high‐elevation sister species, the white‐throated tit, an average of four polymorphisms resulted in five haplotypes averaged across seven loci (Table 2) and haplotype and nucleotide diversity varied between 0.33–0.98 and 0.0003–0.002, respectively. The locus, TLR7, showed a maximum number of polymorphisms and haplotypes for both *Aegithalos* species. Tajima's *D* and Fu and Li's *F* statistic was not significant for any of the loci in both species (Table 2). Fu and Li's *D* statistic was significant for TLR7 in the black‐throated tit (*F* = 1.81; *p* < .05) suggesting an excess of singleton mutations (appearing only once among the sequences), as compared to the total number of mutations. No deviations from HWE were detected in the MAG and CHA populations possibly due to the small sample size (see Table 1 for population code). In the remaining sampling sites, 4–6 loci showed significant deviation from HWE after Benjamini–Hochberg FDR correction (MAN: TLR1LA, TLR2, TLR4, TLR7; ANU: TLR1LA, TLR2, TLR3, TLR4, TLR5, TLR7; KAN: TLR1LA, TLR2, TLR3, TLR4, TLR7; *p* < .05 in all cases). For the black‐throated tit, TLR1LB haplotypes encoded one unique protein whereas the rest of the loci coded between 7–30 unique protein sequences (Table 2 and Table S6). Stop codons were detected in two haplotypes of TLR2 (haplotypes 2 and 3) which were prevalent in 19.2% of the total genotyped black‐throated tit populations. These two haplotypes were present as homozygotes or in combination with each other in nearly half of the samples, indicating a possible overlap of TLR2 function with another locus. In the white‐throated tit, haplotypes from each TLR locus translated between 2–13 unique protein sequences (Table 2). Rarefaction analysis of TLR loci revealed that saturation of the curve was closest for TLR2 and TLR 4 in the white‐throated tit. However, we had not successfully reached an asymptote for the haplotypes recorded in the remaining loci (Table S7).

**TABLE 2:** Characterizing variation at seven TLR loci in the black‐throated tit and the white‐throated tit

Species	Loci	*N*	S	H	H_d_	π	k	P	Tajima's D	Fu and Li's D	Fu and Li's F	Fragment size (bp)	Protein length	ω
Black‐throated tit	TLR1LA	70	13	46	0.94	0.003	3.59	14	1.35	0.87	1.25	1,089	363	0.21
	TLR1LB	78	6	12	0.72	0.001	1.18	1	0.23	1.08	0.94	969	323	0.0001
	TLR2	78	13	26	0.86	0.002	2.09	12	−0.24	−0.43	−0.43	918	306	0.31
	TLR3	72	17	38	0.79	0.005	5.08	12	1.13	−0.18	0.4	1,125	375	0.31
	TLR4	75	12	31	0.92	0.004	2.82	8	0.79	−0.56	−0.08	783	261	0.26
	TLR5	78	7	15	0.56	0.0007	0.8	7	−0.78	1.16	0.58	1,197	399	0.41
	TLR7	77	22	95	0.99	0.004	4.45	30	0.38	1.81^*^	1.49	1,089	363	0.32
White‐throated tit	TLR1LA	6	5	8	0.89	0.002	2.24	5	0.41	0.54	0.59	1,062	354	0.28
	TLR1LB	8	2	3	0.34	0.0004	0.36	3	1.31	1.26	1.44	978	326	NA
	TLR2	8	1	2	0.33	0.0003	0.33	2	−1.04	−0.5	−0.73	981	327	NA
	TLR3	8	3	4	0.44	0.0004	0.48	4	0.16	0.69	0.63	1,104	368	NA
	TLR4	8	1	2	0.5	0.0006	0.5	2	−1.35	−1.12	−1.87	840	280	NA
	TLR5	7	6	7	0.85	0.002	2.01	5	1.31	0.69	1.25	1,227	409	0.41
	TLR7	8	11	13	0.98	0.003	3.41	13	0.24	0.65	0.62	1,188	396	6.24

Abbreviations: *H*, number of haplotypes; *H*_d_, haplotype diversity; *k*, average number of nucleotide differences; NA, ω could not be determined; P, number of unique protein sequences; S, number of segregating sites; *, significance at *p* < .05; π, nucleotide diversity; ω, ratio of non‐synonymous substitution rate (dN) to synonymous substitution rate.

**FIGURE 2:**
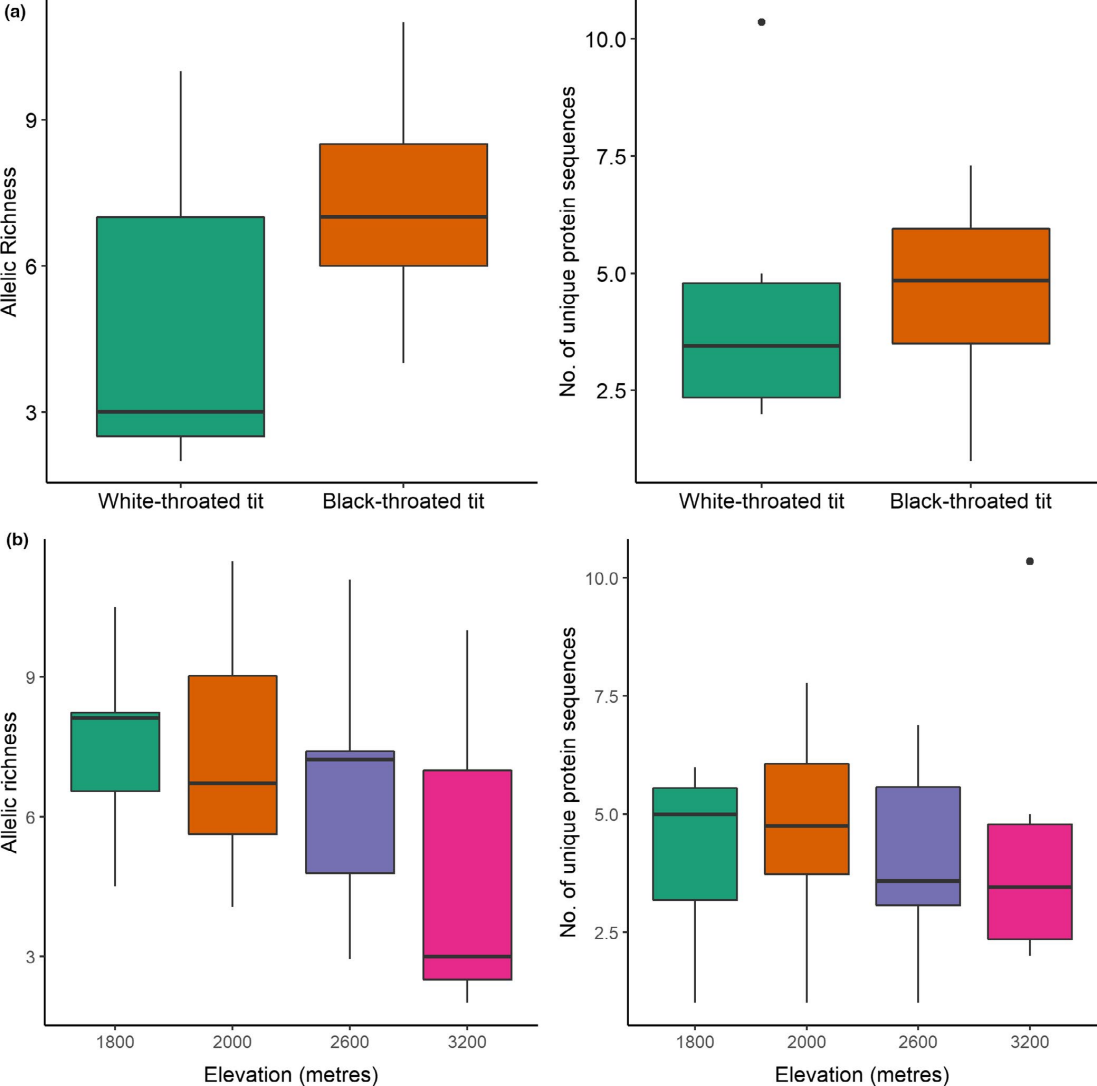
(a) Comparisons of TLR allelic richness (size corrected to *n* = 6) found in the black‐throated tit and white‐throated tit. (b). Comparisons of TLR allelic richness (corrected to *n* = 6) across elevation in the black‐throated tit and white‐throated tit. Here, 1,800, 2,000 m, and 2,600 m refer to low‐, medium‐, and high‐elevation categories in the black‐throated tit and 3,200 m location refer to the white‐throated tit

**FIGURE 3:**
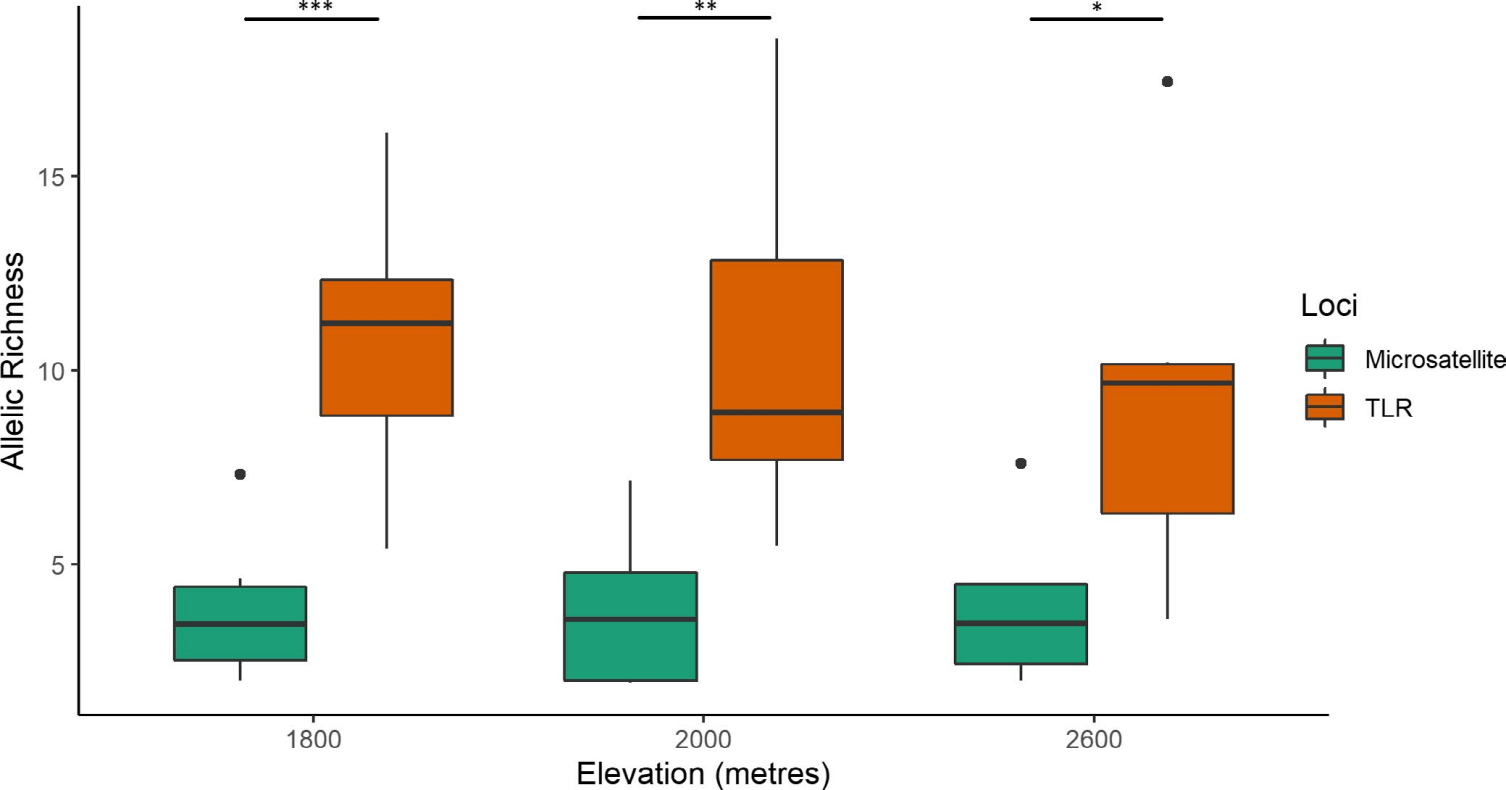
Comparisons of allelic richness (size corrected) using eight neutral loci and seven TLR loci (*p < .05; **p < .01; and ***p < .001) in the black‐throated tit

**FIGURE 4:**
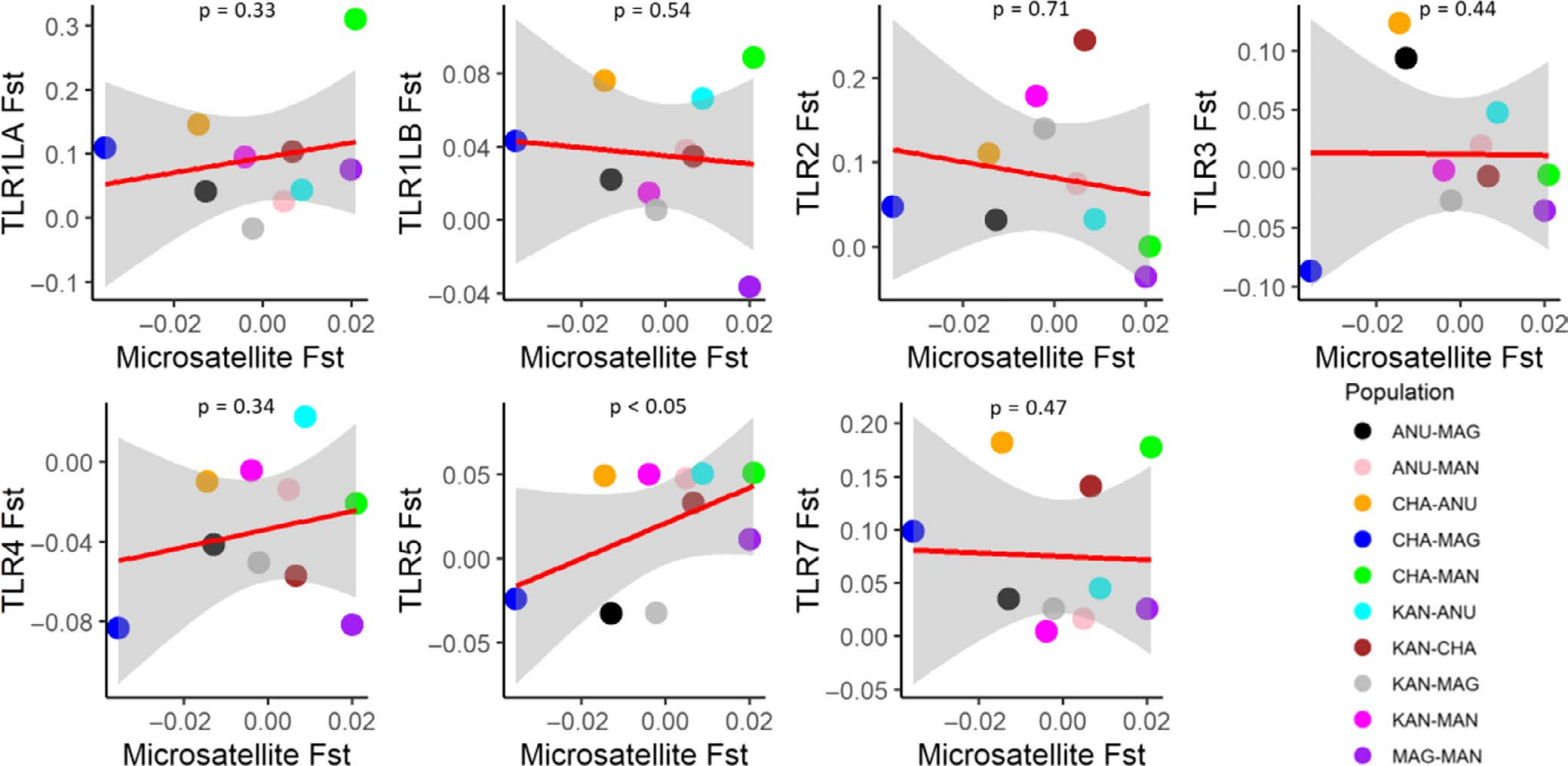
Relationship between pairwise population structure at Toll‐like receptor (TLR) and microsatellite loci among black‐throated tit populations. See Table 1 for population information

Our comparisons of signatures of selection at the haplotype level and the protein level showed no variation in TLR diversity between black‐throated and white‐throated tits (Figure 2a). Comparisons for spatial variation in pathogen‐mediated selection pressure showed no change in TLR diversity across elevations in the black‐throated tit and the white‐throated tit (Figure 2b). However, TLR variation was significantly higher than the neutral variation for the same elevation in the black‐throated tit (Low‐elevation *t* = 4.69, *df* = 9.69, *p* < .001, medium‐elevation *t* = 3.68, *df* = 8.79, *p* < .01, high‐elevation t = 2.87, *df* = 8.49, *p* < .05; Figure 3). Comparison of pairwise *F*_ST_ for neutral and TLR loci showed significant positive association for TLR5 (*r* = 0.49, *p* < .05; Figure 4). We identified between 2–5 clusters for individual TLR locus in the black‐throated tit, with one mutational step from each other (Figures S1–S7). We had too few samples and haplotypes to make a reliable network for the white‐throated tit.

Gene conversion analysis showed that multiple gene conversion events were detected between TLR1LA and TLR1LB in the black‐throated tit (FDR corrected *p* < .05), spanning between a length of 425–609 bp and one event detected in the white‐throated tit, spanning 627 bp.

### 3.2 Pathogen‐mediated selection in TLR genes

ω values using M0 model indicated that TLRs were under purifying selection in both *Aegithalos* species (Table 2). TLR7 showed exceptionally high ω values, indicating positive selection at an overwhelming majority of sites in the white‐throated tit, as identified by both M1a/M2a and M7/M8 models in CODEML, with LRT values significantly differing between the neutral and selection models (*p* < .001 in all cases). In the black‐throated tit, individual codons in TLR1LA, TLR5, and TLR7 were identified to be under positive selection. Some of the codons identified by CODEML were also validated by MEME to be under positive selection at a significance of *p* < .1 (Table 3). The codon identified to be under selection for TLR7 in both species was found to be at a synonymous position using protein alignment.

**TABLE 3:** Positively selected codons identified by the M1a/M2, M7/M8 CODEML models in PAML, and by MEME in the HyPhy package. Functional effect of the amino acid change was predicted by the PROVEAN server

Species	Locus	Positively selected codon	LRT (M1a‐M2a)	LRT (M7‐M8)	LRT (MEME)	Amino acid change	Functional effect
Black‐throated tit	TLR1LA	126	55.95***	57.74***	4.26^***^	T > A	Neutral
	TLR5	345	27.16***	27.37***	4.12^***^	L > S	Deleterious
	TLR7	103	265.41***	258.76***	10.57^***^	E > D	Neutral
White‐throated tit	TLR7	153	77.31***	79.81***	4.26^***^	L > P	Deleterious

*** *p* < .001; ^+^ *p* < .1; ^x^ *p* = 0.

Analysis with GARD identified two recombination breakpoints in all TLR genes in the black‐throated tit and 2–3 breakpoints in all TLR genes of the white‐throated tit (except TLR1LB, TLR2, and TLR3, which could not be tested due to less than three haplotypes).

### 3.3 Spatial variation in TLR haplotypes and infection risk in the black‐throated tit

**FIGURE 5:**
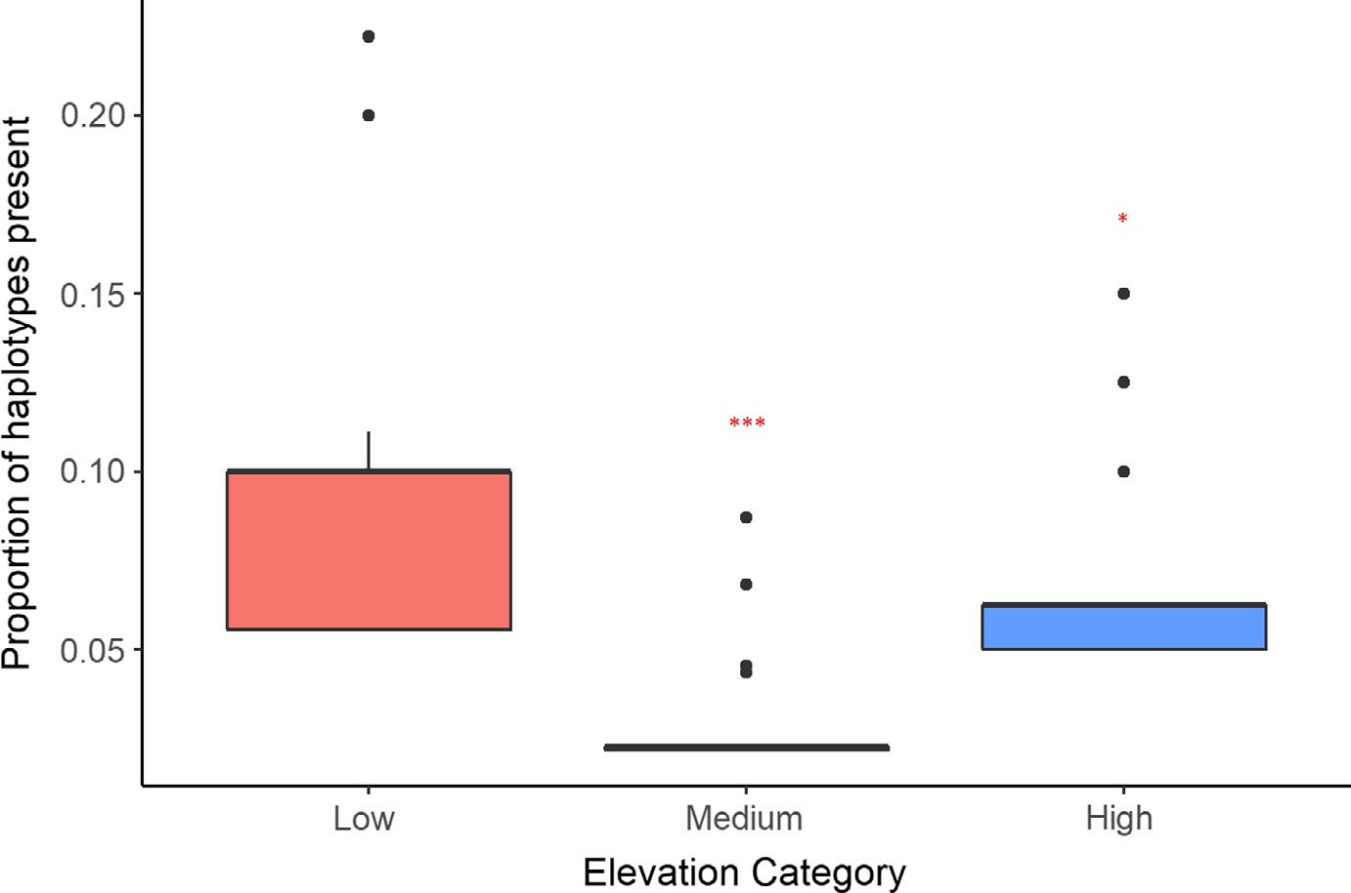
Proportion of TLR7 haplotypes present in the black‐throated tit showed significant variation across elevation using generalized linear model. Significant values are indicated by asterisk (*p < .05; ***p < .001) and in comparison to low‐elevation category

Based on molecular sexing, male (*n* = 40) and female (*n* = 38) birds were nearly equally represented in the black‐throated tit populations. Of the total birds, 24 (30.7%) were infected with avian blood parasites. Of these, 18 (75%) were infected with *Haemoproteus* lineages (AEGCON1 and AEGCON2; Ishtiaq, Bowden, & Jhala, 2017b) at medium elevation (1,800–2,000 m) and 6 (25%) had *Leucocytozoon* infection with three distinct lineages in medium to high‐elevation sites (2,000‐2,600 m). Due to low lineage prevalence, we considered infection status as the predictor variable. TLR7 haplotypes showed significant association with elevation (Wald's χ^2^ = 170.04, *df* = 2, *p* < .0001; Table 4; Figure 5) but no significant effect of sex. However, positively selected codons did not show any significant effect of elevation or sex (Table S8). For clusters and heterozygosity models, infection status was not influenced by any parameter except a negative association with elevation in TLR1LA, TLR3, TLR4 as top model (Table S9).

**TABLE 4:** Number of TLR7 haplotypes in black‐throated tits showed significant association with elevation using generalized linear model

	β	SE	t value	P
Low elevation (Intercept)	−2.31	0.12	19.32	<0.0001
Medium elevation	−1.28	0.12	−10.88	<0.0001
High elevation	−0.28	0.14	−2.04	<0.05
Sex	−0.01	0.09	−0.16	>0.05

### 3.4 Neutral variation in the black‐throated tit

We successfully genotyped 78 black‐throated tit samples, and allele size was determined in the range of 101–343 bp (Table 5 for allele size and Table S10 for genotype information). Of the eleven loci used in this study, three (TG01040, TG04041, and Man13) were monomorphic and were dropped from subsequent analyses (but see Bottleneck analysis). We found no evidence of null alleles or allelic dropout in any of the loci. We found no significant difference in the average observed (*H*_o_ = 0.548) and expected heterozygosity (*H*_E_ = 0.55) across populations and loci (Table 5). PIC values varied from 0.20–0.83, with an average value of 0.50. The mean number of alleles observed across populations and loci was 5.25. All loci were in HWE after Benjamini–Hochberg FDR correction, except Ase18 across all subpopulation and Pca3 for one population (KAN; Table 1). We retained all loci as the subsequent analysis was done across elevation. Pairwise *F_ST_* across locations was found to be very low and varied between 0 and 0.021 (Table S11). Inbreeding coefficient (*F_IS_*) across all loci was low (mean = 0.008; Table 5), indicating the absence of mating between closely related individuals and populations at equilibrium following random mating. The mean inbreeding coefficient for each population was negative, indicating outbreed populations (mean = −0.48; MAN = −0.50, MAG = −0.53, ANU = −0.44, CHA = −0.5, KAN = −0.43) and did not significantly vary across sites, based on the 95% confidence interval (*p* > .05).

**TABLE 5:** Summary of genetic diversity measures for eight polymorphic putatively neutral loci in the black‐throated tit

Locus	*A*_R_	Allele size range (bp)	*H*_O_	*H*_E_	PIC	*F*_IS_ (W&C)
Ase18	7	130–142	0.77	0.71	0.67	−0.08
TG03031	3	208–212	0.19	0.22	0.2	0.12
Pca3	8	137–157	0.64	0.71	0.66	0.1
PmaD22	11	293–343	0.85	0.85	0.83	0
Ase37	3	232–238	0.46	0.52	0.46	0.11
TG04004	2	165–167	0.45	0.36	0.3	−0.23
Escu6	6	101–113	0.59	0.55	0.51	−0.07
TG01147	2	277–281	0.44	0.49	0.37	0.11

Abbreviations: *A*_C_, Allelic richness corrected for size (*n* = 6); *A*_R_, Allelic richness; *F*_IS_ (W&C), Weir and Cockerham inbreeding co‐efficient; *H*_E_, Expected Heterozygosity; *H*_O_, Observed Heterozygosity; PIC, Polymorphism Information Content.

### 3.5 Population genetic structure and gene flow in the black‐throated tit

**FIGURE 6:**
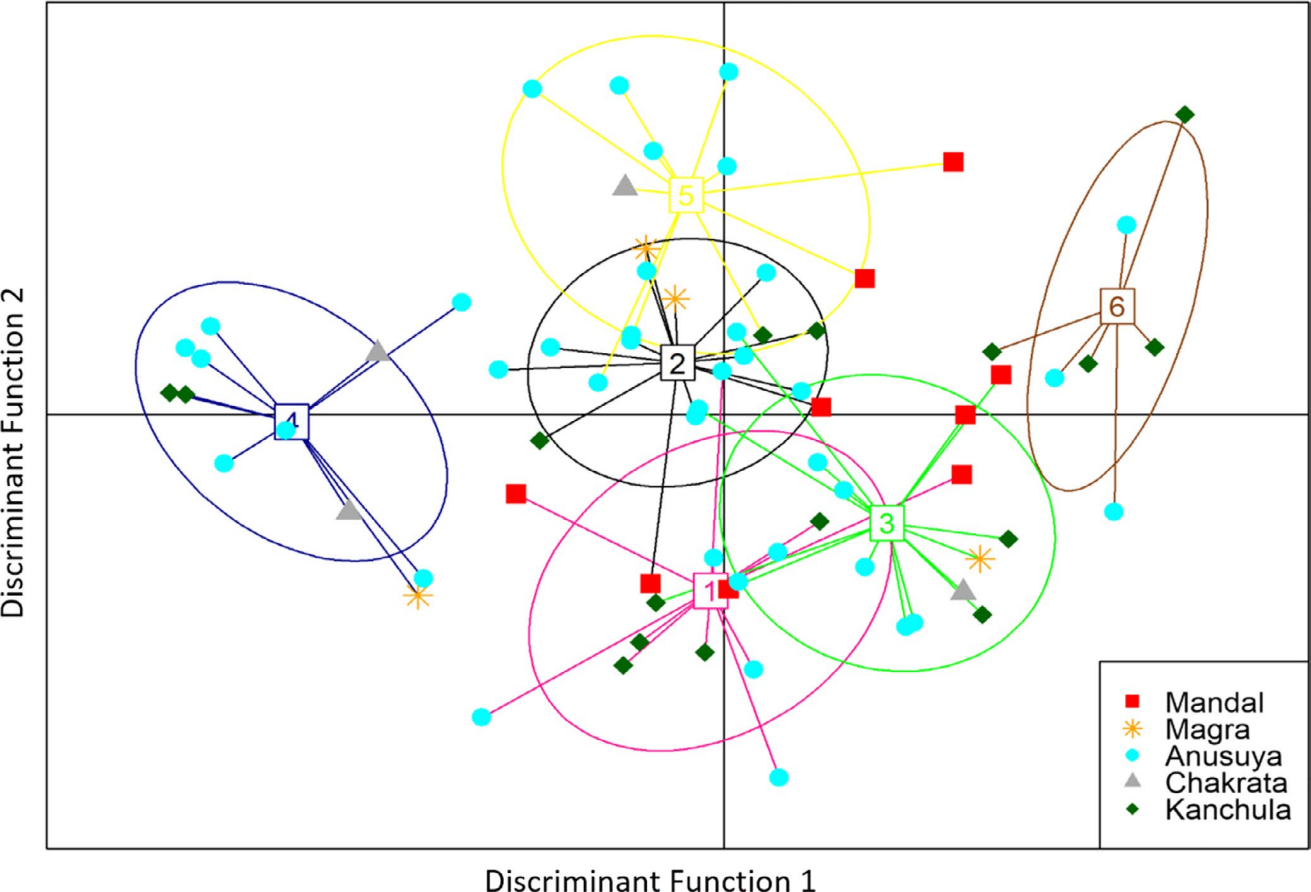
Genetic structure analysis by DAPC using eight putatively neutral loci showed the presence of six genetic clusters in the black‐throated tit. Samples from all locations are distributed among the different clusters and no sequestration of sampling sites is visible

STRUCTURE analyses within species indicated seven clusters (*K* = 7) with weak genetic structure and high gene flow between populations (Figure S8). DAPC analysis indicated six genetic clusters with considerable overlap among four clusters (Figure 6), as well as a lack of segregation by sex (Figure S9). Nearly 88% of the individuals could be categorized with >70% membership probability to one of the six different clusters. We considered four groups for AMOVA: (a) DAPC clusters 2 and 5; (b) clusters 1 and 3; (c) cluster 4; (d) cluster 6 (Figure 6 for DAPC cluster details). We found 13.84% of variance observed was attributable to significant differences in populations within groups (*p* < .001) whereas 4.25% with no significant difference at the group level (*p* > .05; Table S12). The Mantel test showed no evidence of any decrease in genetic differentiation with an increase in geographic distance across sampled populations (*r* = 0.004, *p* = .46; Figure S10).

### 3.6 Comparison of genetic variation in the black‐throated tit across two regions: India and China

#### 3.6.1 Spatial autocorrelation

We found no relationship between relatedness and spatial distance between pairs of individuals in India (*b* = −0.0005, SE = 0.0004; *p* > .05; Figure S11a), and a significant negative relationship (*b* = −0.04. SE = 0.001; *p* < .001; Figure S11b) between pairs of individuals in China.

#### 3.6.2 Signatures of bottleneck

The *M*‐ratio of allelic richness to allelic size range was low for both India and China populations, indicative of a past bottleneck, whereas tests of heterozygosity excess suggested that only China populations have experienced any recent bottlenecks (Table 6)—the mode distribution curve for both India and China populations followed an L‐shaped curve indicating no bottleneck and adherence to drift–mutation equilibrium.

**TABLE 6:** Results of genetic bottlenecks analysis using eleven microsatellite loci: recent (TPM, SMM) and ancient population bottleneck (M‐ratio)

Dataset	Bottleneck			M‐ratio
	Significance test	TPM	SMM	Modified Garza–Williamson index
India (this study)	Sign test	0.52	0.24	0.15
	Wilcoxon signed‐rank test (two‐tailed)	1	0.46	
China (Dai et al., 2017)	Sign Test	0.034^*^	0.007^**^	0.39
	Wilcoxon signed‐rank test (two‐tailed)	0.012^*^	0.005^**^	

Note

China data on the black‐throated tit used were 78 randomized samples. SMM: stepwise mutation model; TPM: two‐phase model; * *p* < .05; ** *p* < .01. Distribution of the mode was found to be L‐shaped for both populations in BOTTLENECK, indicating an absence of bottleneck event.

## 4 DISCUSSION

We characterized genetic variation in neutral and adaptive loci in a widespread Himalayan passerine, the black‐throated tits, which showed very low genetic diversity, absence of genetic structure, and evidence of a past genetic bottleneck. By contrast, diversity in TLR loci was high and exhibited locus‐specific (TLR1A, TLR5, and TLR7) signatures of selection; however, there was no evidence of selection driven by malaria although this could be influenced by some other pathogens. Furthermore, we quantified variation in adaptive loci to understand selection pressure at three levels: (a) there was no change in TLR diversity within species across an elevational gradient, (b) there was no association in spatial variation in TLR diversity with infection risk, and (c) comparisons with a high‐elevation sister species exhibited patterns of selection pressure shaping diversity at a specific TLR locus.

### 4.1 TLR variation in two Aegithalos species

Across the seven TLR loci, we found 12–95 alleles in the black‐throated tit while we found 2–14 alleles in the white‐throated tit. These levels of TLR variation are remarkably higher than those reported for recently bottlenecked species (New Zealand robin, Grueber et al., 2012; Berthelot's pipit, Gonzalez‐Quvedo et al., 2015; African penguin, Dalton et al., 2016; Chinese egret, Xu et al., 2020) and is more comparable to those reported in widespread species (e.g., lesser kestrel, house finch, Alcaide & Edwards, 2011). We did not find contrast in TLR variation between two species. While our sample size for each elevation category was probably low to detect the selection, the levels of variation in TLR loci were relatively similar in the two sister species. Furthermore, we found no variation between the allelic richness or number of protein sequences for each elevational category in either the black‐throated tit (low, medium, and high) or the white‐throated tit. This suggests that both *Aegithalos* species experience a mixture of ecological and evolutionary processes that influence variation in orthologous immune genes in similar ways. The extent of species replacement across an elevational gradient in the western Himalaya is largely driven by habitat heterogeneity and environmental filters. While the distribution range and habitat use in two *Aegithalos* species do not overlap, these are ecologically similar, congeneric species. We believe that a similar pathogenic burden operates across elevation and distribution range of such species (e.g., *Leucocytozoon*; Ishtiaq & Barve, 2018). Furthermore, we detected a common positively selected codon in TLR7 from both *Aegithalos* species. This is discernible from the observation that TLR7 of the white‐throated tit was positively selected, where a high ω value (high nonsynonymous substitution rate) was noted. Additionally, while TLR7 haplotypes show a decrease in distribution with elevation, this trend was not extendable to the white‐throated tit, where TLR7 allelic richness increased. Nonetheless, the high TLR genetic diversity displayed in both *Aegithalos* species is indicative of a robust immune system that can perhaps withstand the pressure of existing and potentially new parasites. The high TLR7 haplotype diversity observed in our data should however be treated cautiously as the gene has been shown to be duplicated independently in certain passerine lineages (Velová et al., 2018), although we had little proof to suspect duplication from chromatogram peaks.

### 4.2 TLR diversity and infection status

While elevation‐dependent decrease of certain pathogens has been documented (e.g., *Borrelllia*, Cornetti et al., 2018; *Plasmodium* and *Haemoproteus*, Ishtiaq & Barve, 2018), it is possible that high‐elevation pathogens (e.g., *Leucocytozoon*; Ishtiaq & Barve, 2018) impose a uniform or possibly higher pathogenic burden on certain loci in western Himalayan birds. Given the low sample size in the white‐throated tit, the prevalence of *Leucocytozoon* infection has been nearly absent and no *Haemoproteus* or *Plasmodium* infections which is in line with infection patterns observed in high‐elevation birds (Ishtiaq & Barve, 2018). The black‐throated tit harbored two host‐specific *Haemoproteus* lineages (AEGCON1 and AEGCON2; Ishtiaq et al., 2017a) and three distinct *Leucocytozoon* lineages. Studies in other taxa prove a clear relationship between infection status and specific TLR haplotypes (Cornetti et al., 2018; Kloch et al., 2018; Tschirren et al., 2013). A recent study established association of allele frequencies at three TLR loci (TLR1LA, TLR2B, and TLR7) with infection of malarial parasites in songbirds (Antonides et al., 2019). We were not able to establish a similar relationship in our system using malaria infection data, possibly due to low numbers of infected individuals recorded and low sample size. One possible explanation for balancing selection is that of heterozygote advantage, where heterozygous states are better suited to combat a wider variety of pathogens (Doherty & Zinkernagel, 1975 but see Ejsmond & Radwan, 2015; Esjmond et al., 2014; Spurgin & Richardson, 2010). However, we were unable to find associations between infection status and heterozygosity as well.

### 4.3 Neutral versus TLR variation in the black‐throated tit

The variation in TLR loci was significantly higher than neutral loci. We detected high diversity in TLR loci in the black‐throated tits. The significant differences in genetic diversity between neutral and adaptive markers showed the influence of pathogen‐mediated selection in TLRs. Studies on bottlenecked populations demonstrated that TLR diversity was nearly the same (Gilroy et al., 2017; Gonzalez‐Quevedo et al., 2015), or even less diverse than neutral loci (Hartmann et al., 2014). Both neutral and functional genetic diversity are affected by random fluctuations in the number of alleles due to genetic drift, population bottleneck, demographic history, and gene flow in a population. There is no data on the population size and demographic histories of the black‐throated tit. Given that TLR diversity remained high even after a bottleneck event, we found two possible signatures of the influence of population bottleneck on TLR loci. First, pairwise *F*_ST_ comparisons of individual TLR loci with neutral markers revealed that TLR5 correlated positively with *F*_ST_ values of microsatellites. This indicated that both TLR5 and microsatellites were affected by similar evolutionary processes. Second, there was low prevalence of two TLR2 stop codon haplotypes in the black‐throated tit populations. Bottleneck events in adaptive loci can lead to rapid fixation of rare alleles in a population and this can sometimes be disruptive, as it may lead to the fixation of deleterious alleles in the population (Lande, 1988, 1994). The presence of these stop codon haplotypes could have long‐reaching negative biological consequences, by disruption of protein function resulting in increased susceptibility to certain infections (Hawn et al., 2003, 2005; Merx et al., 2007).

Determining the role of selection on TLR diversity is however complicated by the presence of recombination sites in all TLR genes. Recombination events can significantly influence genetic diversity in immune genes such as MHC (Wutzler et al., 2012), but this is less common in TLRs (Alcaide & Edwards, 2011; Fisher et al., 2011; Gilroy et al., 2017; Gonzalez‐Quevedo et al., 2015) and therefore warrants further investigation. Furthermore, in line with previous observation (Velová et al., 2018), we found conversion events between the paralagous genes TLR1LA and TLR1LB, that could account for the high diversity observed.

Selection pressure on TLR loci is predominantly expected to be negative, where only synonymous mutations are favored, resulting in no increase in genetic diversity (Alcaide & Edwards, 2011; Shultz & Sackton, 2019). Patterns of selection in other taxa (e.g., mammals) implicate balancing selection as one of the forces of evolution maintaining variations in TLR loci (Ferrer‐Admetlla et al., 2008; Kloch et al., 2018), although this has been less explored in birds. We observed mixed patterns of selection on different TLR loci in our study, providing insights into their functional importance. For example, TLR1LB was found to be under strong purifying selection, indicating a conservation in function. The higher nucleotide diversity in TLR loci as compared to protein sequence diversity signifies a functional constraint in TLR proteins and validates the observation that most sites in TLR genes are under purifying selection. A marked departure in our observation was the detection of positively selected codons in TLR7 from both *Aegithalos* species, a loci found to be under strong purifying selection in other studies (Alcaide & Edwards, 2011; Shultz & Sackton, 2019).

### 4.4 Lack of genetic diversity, population structure, and bottleneck

The western Himalayan populations of the black‐throated tit were panmictic and genetically homogeneous. The Li et al. (2012) study on the black‐throated tit populations in China showed that natal dispersal distances are limited to 5 km which is probably enough to maintain connectivity at a small spatial scale. Our findings are in contrast with a study on a similar spatial scale (150 km) in China showing population differentiation with increase in geographic distance (Dai et al., 2017). The black‐throated tit is morphologically diverse and forms genetically distinct lineages, which have recently split as separate species as *A. iredalei* in India and *A. concinnus* in China. Of the eleven neutral loci used, three loci were monomorphic in India populations, however, displaying moderate levels of heterozygosity (0.2–0.6) relative to China. The black‐throated tits sampled in China were two lineages (5% divergence), *A. c. concinnus,* and *A. c. talifuenses*, with a high level of gene flow—and have been merged as a single species (Dai et al., 2017). The relatively high genetic variation in China could be attributed to hybridization between two lineages with male‐biased gene flow. While we found no relationship between inbreeding coefficient and geographic distance in India, the neighboring spatially close (5 km) individuals were more genetically related than any random individual in the 20 km distance class of China populations. Absence of a significant pattern in India could be attributed to fragmented habitat recently colonized by a few individuals with reduced genetic diversity.

The drift–selection equilibrium can be tipped in the favor of drift influencing diversity and is prominently felt in small or reduced populations, such as those experiencing a population bottleneck (Gonzalez‐Quevedo et al., 2015). Our bottleneck analyses on two regional populations showed contrasting results—the Indian population exhibited signatures of a past bottleneck whereas the China population showed a heterozygote excess as measured by two mutation models, indicating a population expansion (Cornuet & Luikart, 1996), which is in line with observations from previous studies (Dai et al., 2011). In the absence of sufficient ecological and population historical data, we cannot determine the exact timing or estimate the extent of the bottleneck event. A bottleneck was detected in the Indian population using *M*‐ratio but the lack of heterozygosity excess shows a signature of old but severe bottleneck with a subsequent recovery. It is possible that the black‐throated tit population declined in the distant past due to habitat disturbance such as forest fires or logging. Uttarakhand forests have been exposed to major forest fires every year (Chitale & Behera, 2019; Gupta et al., 2018) which could lead to fragmented habitat and influence genetic patterns and structure, local extinctions, and subsequent colonization.

In conclusion, our study shows that even though a past bottleneck event altered variations in neutral loci, diversity in most TLR genes was relatively high despite the effects of genetic drift. Measures of functional gene diversity provide critical information in identifying vulnerable species, as reduced TLR diversity would hint at a lowered ability to recognize new or diverse pathogens, ultimately affecting individual fitness (Dalton et al., 2016). TLR diversity in the black‐throated tit is high, in contrast to observations from several endangered species (Dalton et al., 2016; Gilroy et al., 2017), indicating that this species is possibly not subject to any severe threat from disease. However, the white‐throated tit warrants further investigation with robust sample size. In black‐throated tits, evolution of TLRs seemed to be slightly more complex as compared to similar studies in other birds, with evidence of recombination, gene conversion, purifying, and positive selection observed (Alcaide & Edwards, 2011). Improved sampling size and comparative analysis with other adaptive loci such as MHC can provide deeper insights into the evolutionary pressure acting on immune genes. This is especially important in an environment of altering pathogenic burden caused by climate change, leading to the range expansion of parasites. Such studies can eventually prove valuable for understanding immunogenetics in migratory birds, where drastic changes in the climate, geography, and environment can affect ability to combat infections.

## CONFLICT OF INTERESTS

No competing Interests.

## AUTHOR CONTRIBUTION

**Farah Ishtiaq:** Conceptualization (lead); Data curation (supporting); Formal analysis (supporting); Funding acquisition (lead); Investigation (lead); Methodology (lead); Project administration (lead); Resources (lead); Software (lead); Supervision (lead); Validation (lead); Writing‐review & editing (lead). **Mridula Nandakumar:** Data curation (supporting); Formal analysis (lead); Methodology (supporting); Software (equal); Writing‐original draft (supporting); Writing‐review & editing (supporting).

## AUTHOR CONTRIBUTIONS

FI: Idea and design of the experiment. FI: Field and MN laboratory experiments. MN: Data analysis. FI and MN: Manuscript writing. Both authors approved the final version of the manuscript.

## Supporting information

Fig S1‐S11

Table S1‐S12

## Data Availability

All data are available in the Table S2. DNA sequences with accession number MN629487‐MN646077 are submitted in GenBank database.
