# Supplementary material for: Genetic drift and bottleneck do not influence diversity in Toll‐like receptor genes at a small spatial scale in a Himalayan passerine
Source: Ecol Evol. 2020 Oct 15;10(21):12246–63. doi: 10.1002/ece3.6855 (PMC7663051; doi:10.1002/ece3.6855)

**Supplementary Figures:**

**Genetic drift and bottleneck do not influence diversity in toll-like receptor genes at a small spatial scale in a Himalayan passerine**

Figures S1-S7: Haplotypye networks for all TLR loci in the black-throated tit, using minimum spanning trees. Node sizes are proportionate to the distribution of haplotypes. Number of mutations between two nodes are indicated along the edges.

**S1- TLR1LA:**


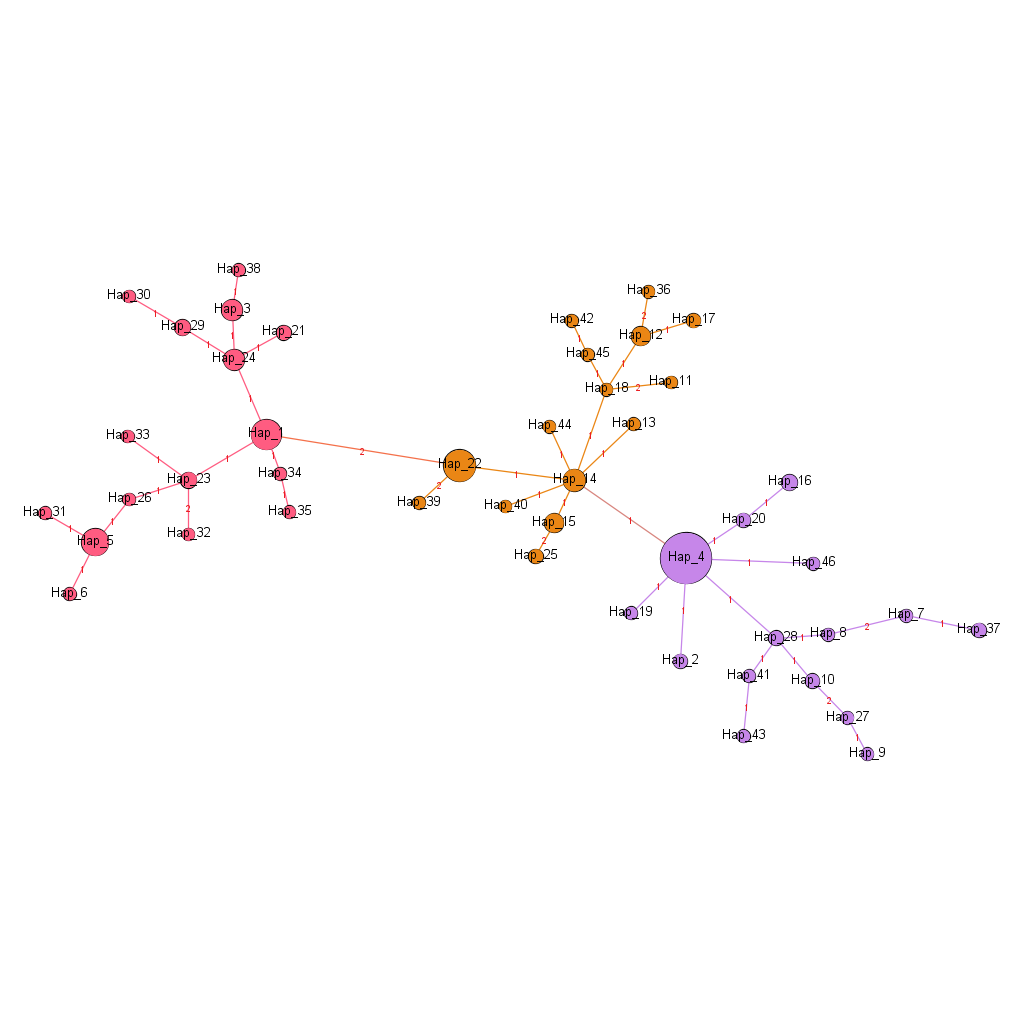


**S2 – TLR1LB**


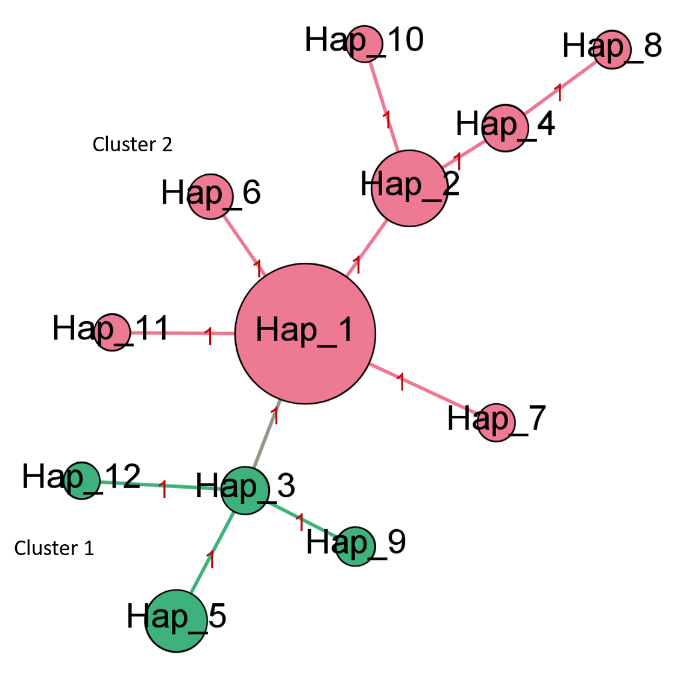


**S3 – TLR2:**


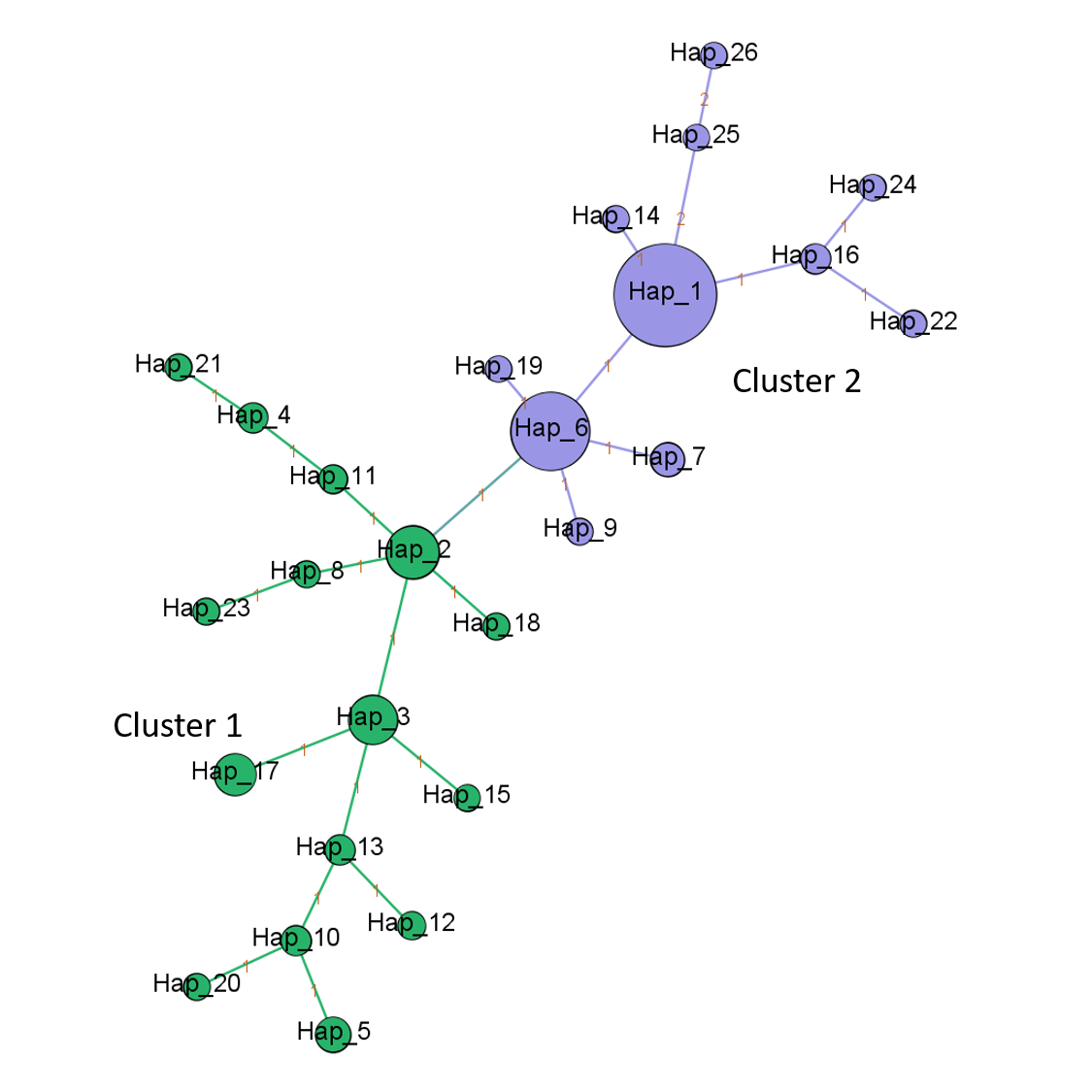


**S4: TLR3**


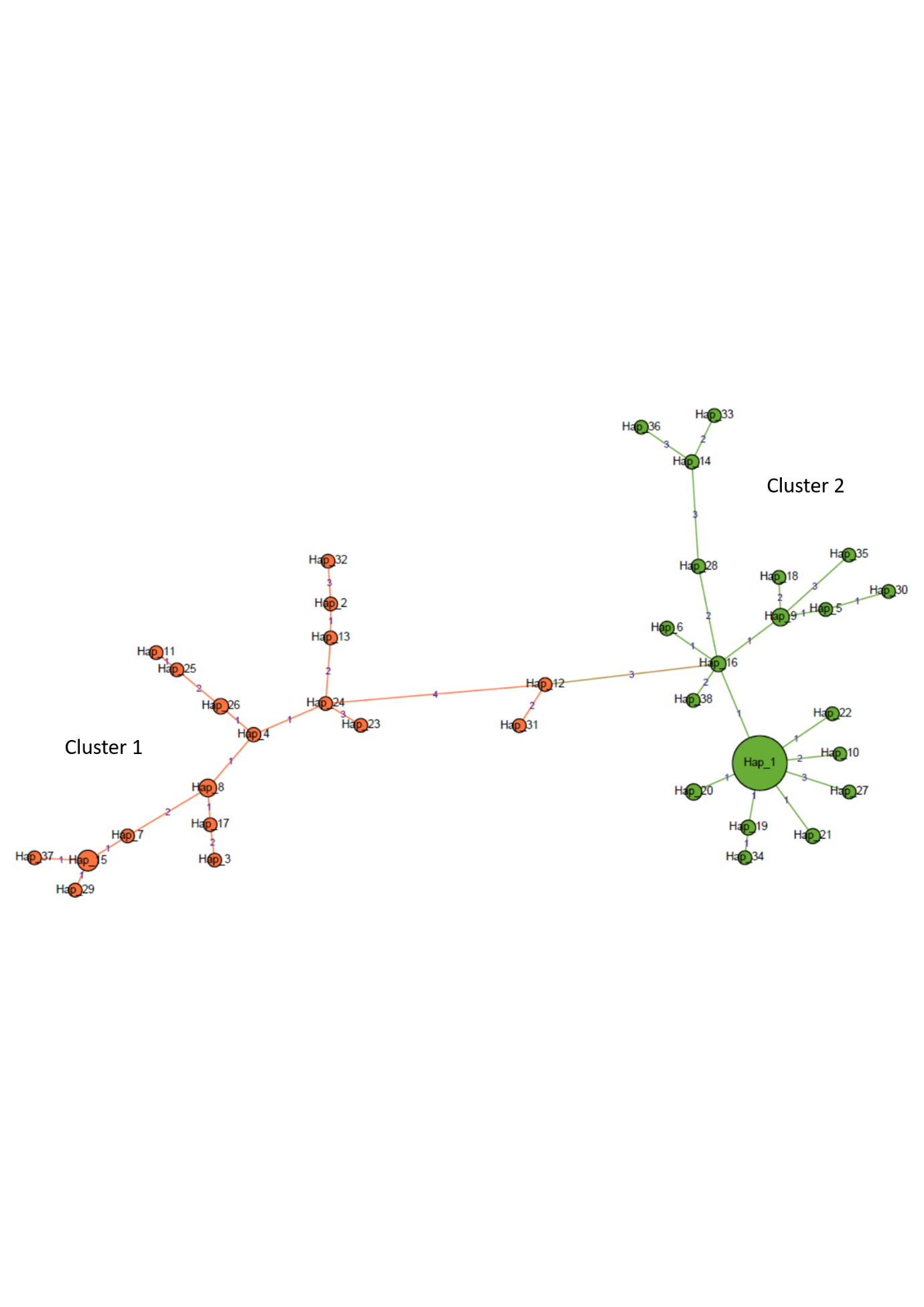


**S5: TLR4**


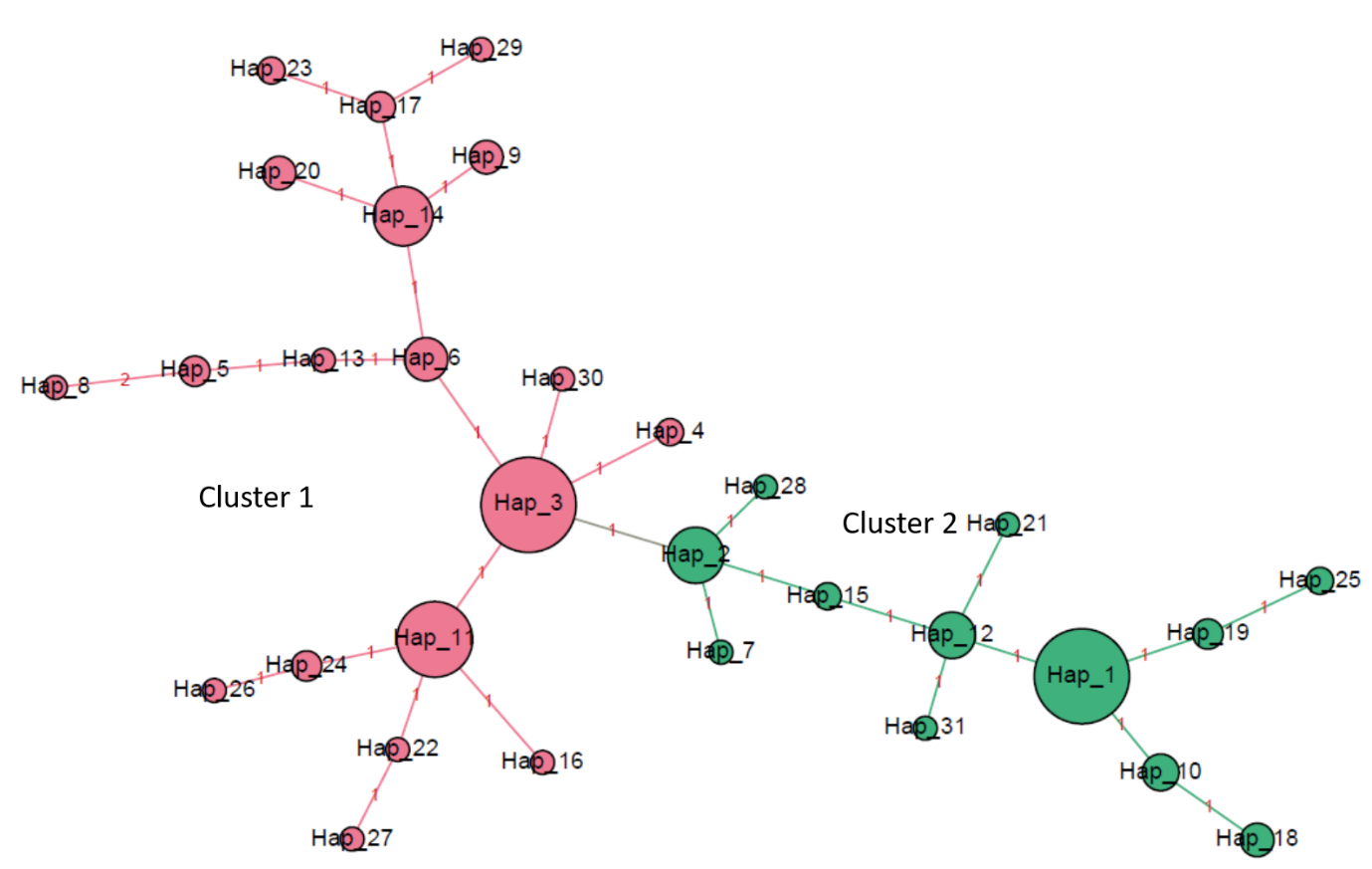


**S6: TLR5**


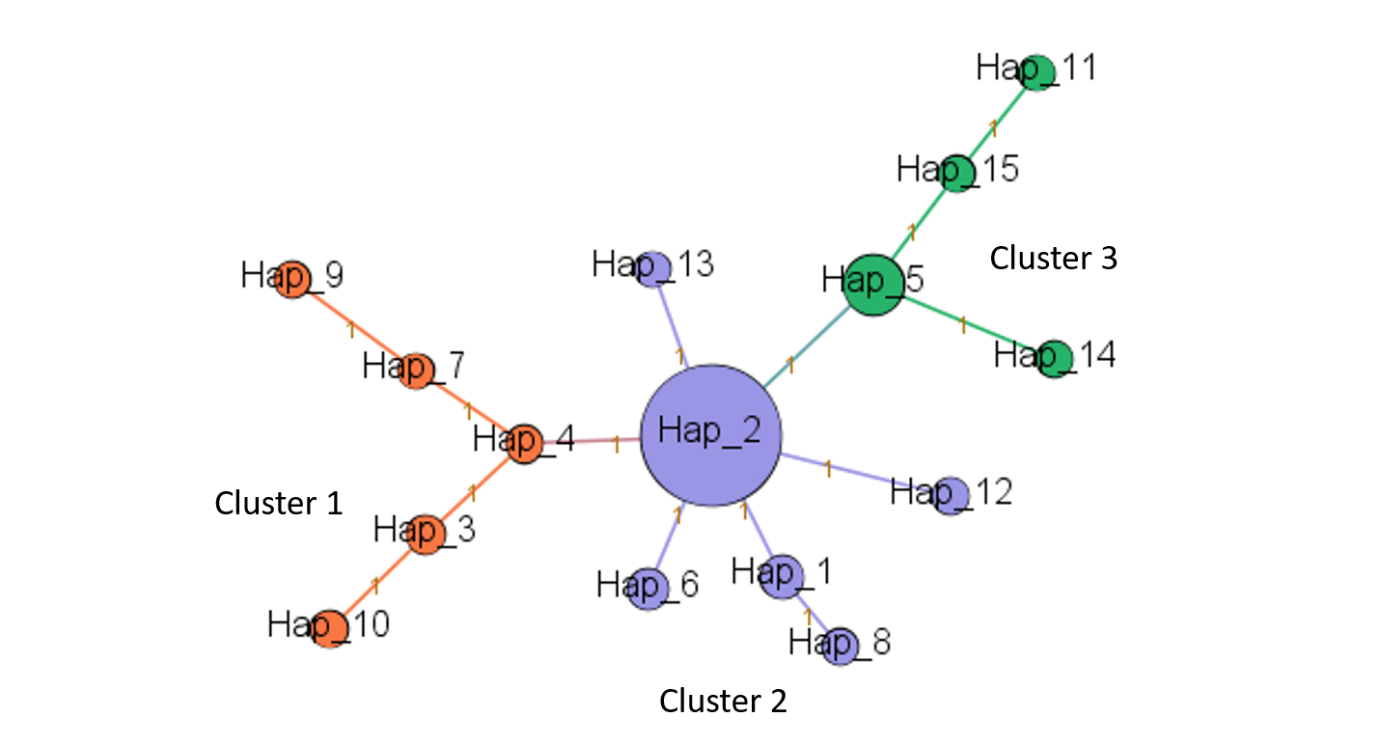


**S7 – TLR7:**


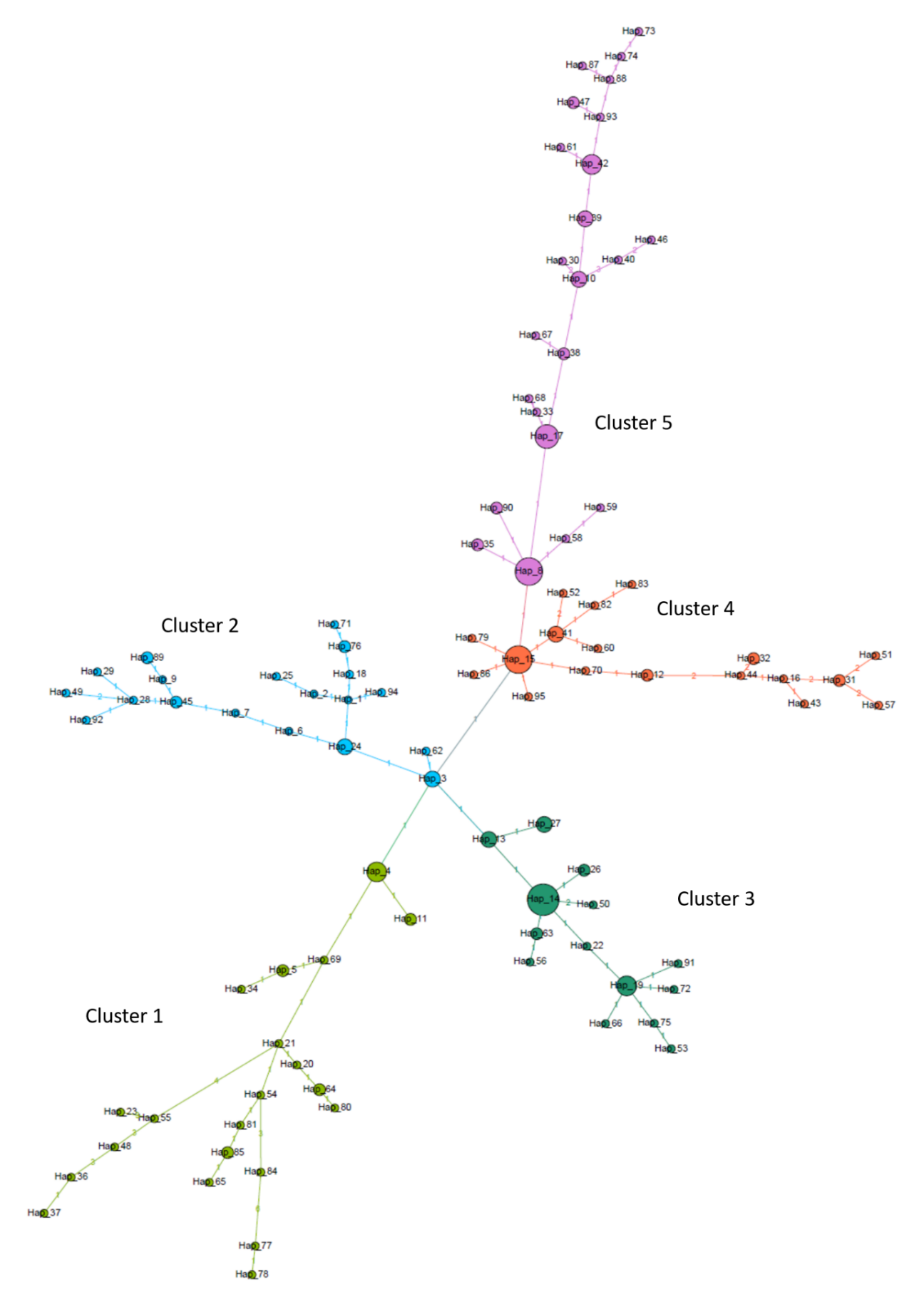


Figure S8a): STRUCTURE analysis showed that the expected number of clusters in the black-throated tit populations was K=7 based on DeltaK values. S8b) STRUCTURE plot suggests high gene flow between populations
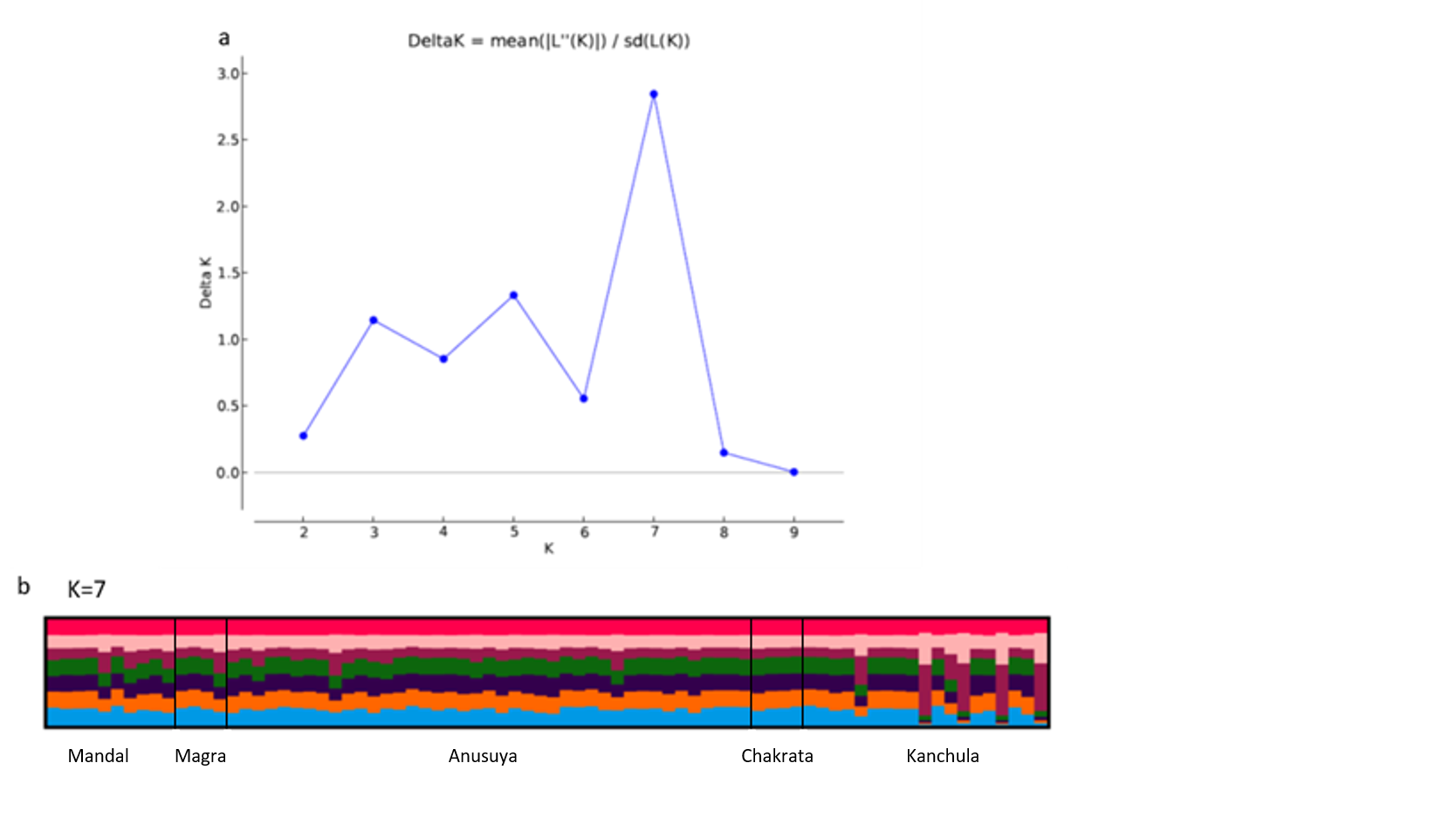


Figure S9: Absence of genetic clusters by DAPC with individuals highlighted by sex.


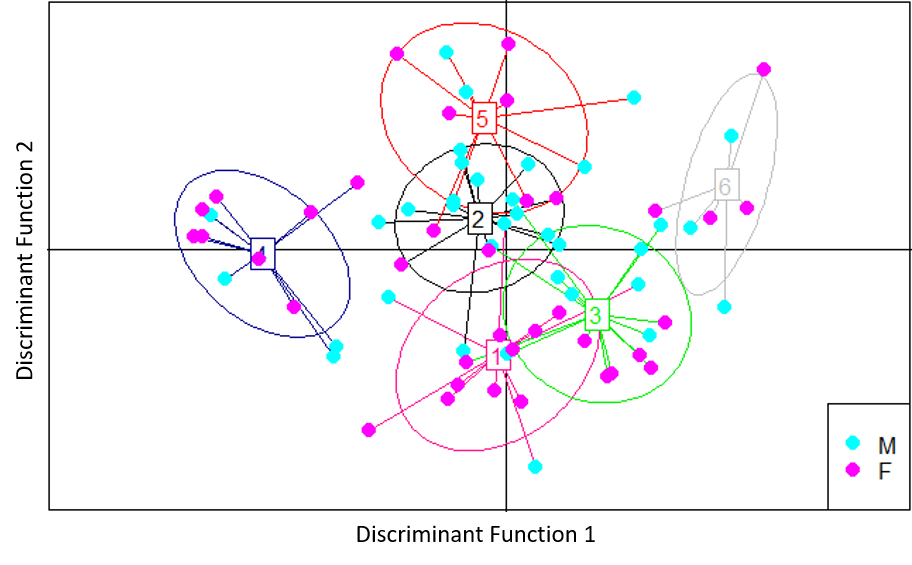


Figure S10: Regression of Slatkin’s linearized *F_ST_* with log geographic distance was not signficant for identifcation of isolation by distance


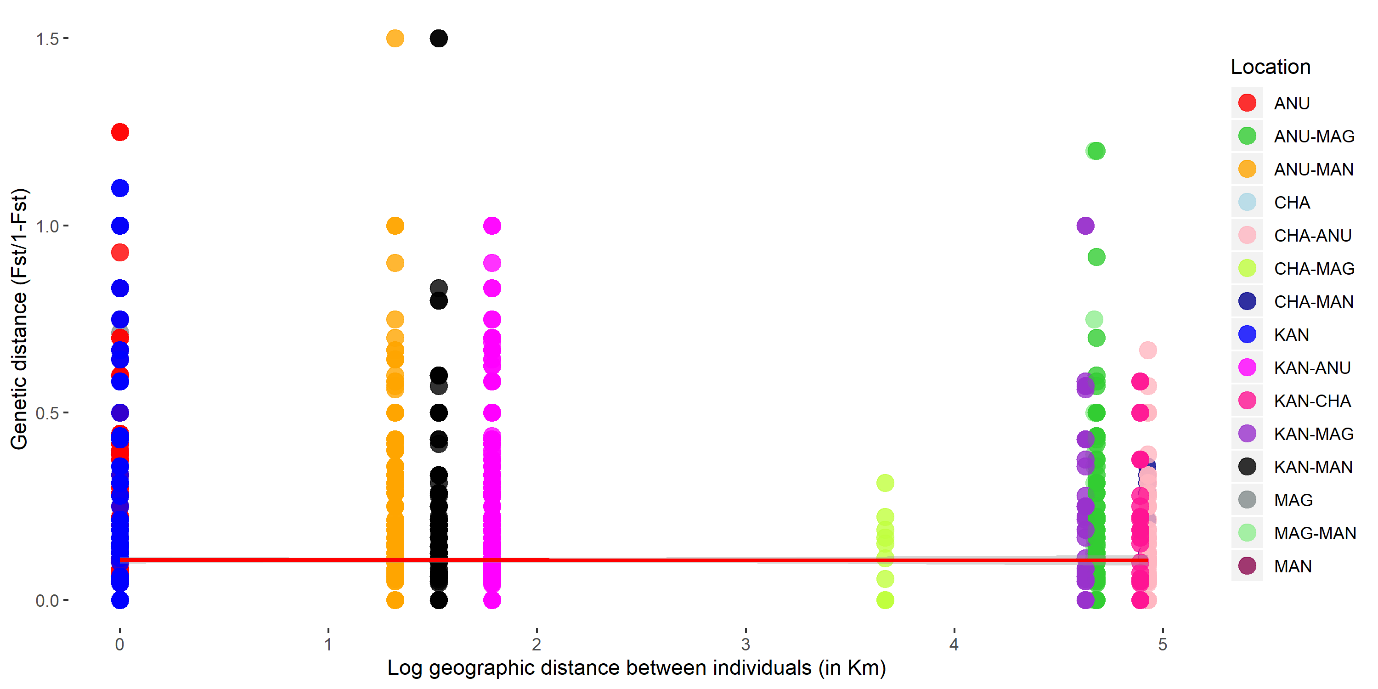


Figure S11: Spatial autocorrelation using kinship coefficient *F_ij_* for: a) India data did not show relatedness with distance unlike b) China data where relatedness reduced with distance.


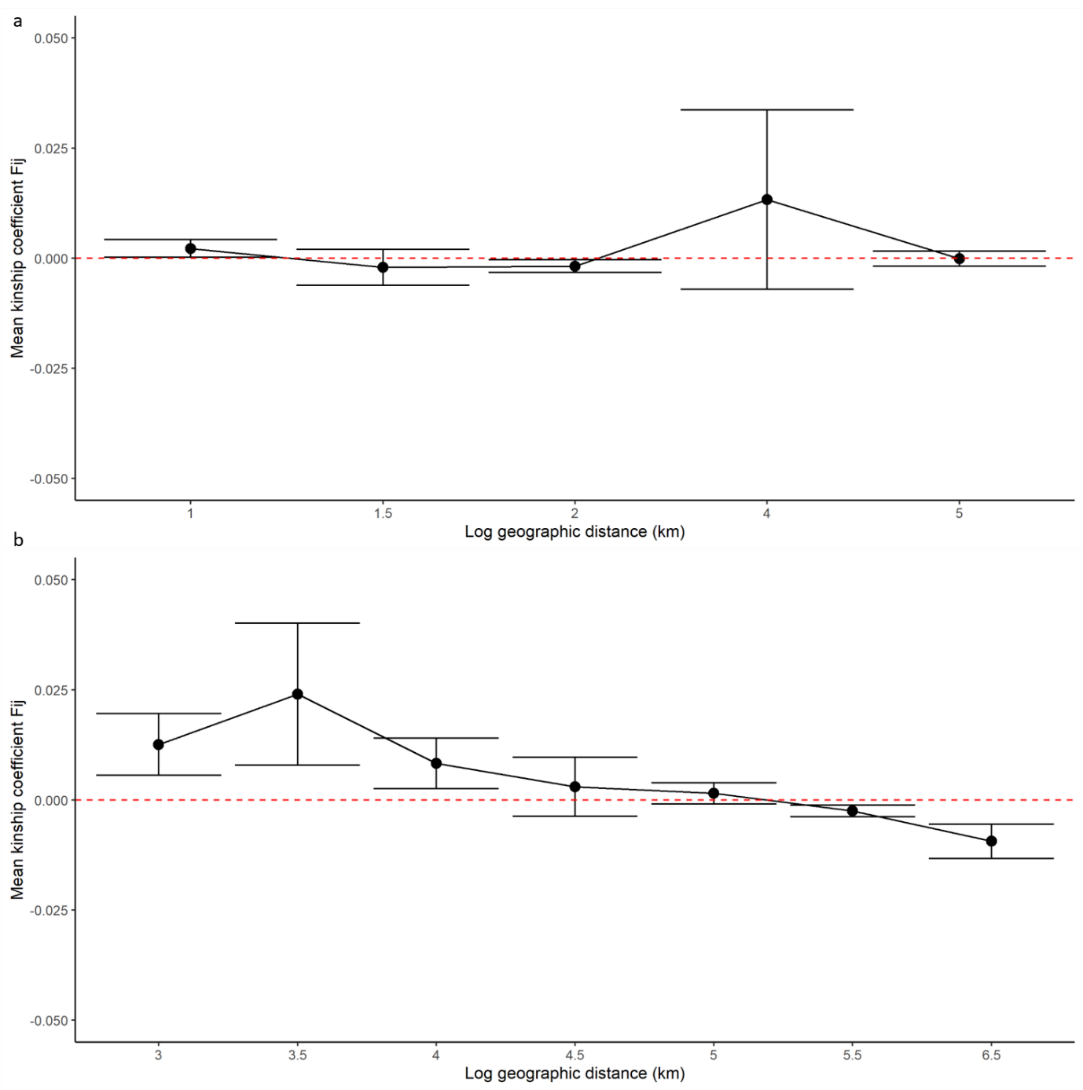

Supplement: Supplementary file 1 — Fig S1‐S11 [file ECE3-10-12246-s001.docx]
